# MET signaling drives acquired resistance to erdafitinib in muscle-invasive bladder cancer cells

**DOI:** 10.1038/s41419-025-08221-8

**Published:** 2025-11-28

**Authors:** Syunta Makabe, Kyoka Hoshi, Hiromi Kaneko, Yusuke Masuishi, Kei Yaginuma, Satoru Meguro, Akifumi Onagi, Seiji Hoshi, Junya Hata, Hiromi Ito, Hiroki Shimura, Yoshiyuki Kojima, Michiru Nishita

**Affiliations:** 1 Department of Urology, Fukushima Medical University School of Medicine, Fukushima, Japan; 2 Department of Biochemistry, Fukushima Medical University School of Medicine, Fukushima, Japan; 3 Department of Laboratory Medicine, Fukushima Medical University School of Medicine, Fukushima, Japan; 4 Department of Hygiene and Preventive Medicine, Fukushima Medical University School of Medicine, Fukushima, Japan

**Keywords:** Urological cancer, Cancer therapeutic resistance

## Abstract

Muscle-invasive bladder cancer (MIBC) is an aggressive malignancy with high recurrence and poor survival, accounting for the majority of bladder cancer–related deaths. A subset of MIBC harbors *FGFR1* amplification or overexpression, associated with increased proliferation and poor prognosis. Although the pan-FGFR inhibitor erdafitinib has demonstrated clinical benefit in patients with *FGFR3/FGFR2* alterations, primarily in non-MIBC, its efficacy is limited by resistance and toxicity. Moreover, its effectiveness in *FGFR1*-driven MIBC remains unclear. To address this gap, we investigated erdafitinib response and resistance mechanisms in JMSU1 cells, a model of *FGFR1*-amplified MIBC. While erdafitinib initially suppressed tumor growth, prolonged treatment led to resistance, characterized by persistent activation of ERK, AKT, and STAT1 signaling pathways. Mechanistic studies identified MET activation, driven by *MET* gene amplification, as a key driver of resistance. Notably, exogenous hepatocyte growth factor (HGF) not only induced resistance but also accelerated the emergence of *MET*-amplified, HGF-independent subpopulations under drug pressure. We also identified SHP2 as a critical mediator of FGFR1-driven ERK activation in parental cells. In resistant cells, MET activation enhanced SHP2–ERK signaling through the adaptor protein GAB1, reinforcing the resistant phenotype. Combined inhibition of FGFR1 and MET significantly suppressed tumor growth in resistant cells. These findings establish *MET* amplification and GAB1–SHP2 signaling as central mediators of erdafitinib resistance in *FGFR1*-amplified MIBC and support dual FGFR1/MET targeting as a promising therapeutic strategy.

## Introduction

Bladder cancer remains a significant global health challenge, responsible for over 200 000 deaths in 2020 [1]. Most cases are urothelial carcinomas, classified into non-muscle-invasive (NMIBC, ~75%) and muscle-invasive bladder cancer (MIBC, ~25%). While NMIBC is confined to the urothelium (stages Ta–T1), MIBC (stages T2–T4) is more aggressive, with a higher risk of metastasis and poor prognosis. High-grade T1 NMIBC often progresses to MIBC despite treatment, complicating disease management [2]. MIBC accounts for the majority of bladder cancer–related deaths, with recurrence or metastasis in up to 50% of cases and five-year survival rates below 50% [3].

Fibroblast growth factor receptors (FGFRs) play a central role in bladder cancer pathogenesis [4, 5]. FGFR1–4 regulate key cellular processes including proliferation, differentiation, and angiogenesis [6], and their dysregulation activates oncogenic signaling pathways such as MAPK, PI3K-Akt, and STATs [7]. *FGFR3* mutations and fusions are common in NMIBC, particularly in low-grade, non-invasive tumors [8], and have led to the development of FGFR-targeted therapies. The pan-FGFR inhibitor erdafitinib, approved by the FDA, provides clinical benefit in *FGFR3*- or *FGFR2*-altered urothelial carcinoma. In the phase 3 THOR trial (Cohort 1), erdafitinib significantly improved median overall survival (12.1 vs 7.8 months) and objective response rate (45.6% vs 11.5%) over chemotherapy, but the median duration of response remained short (~4.9 months overall, and ~4.9–6.7 months across populations in related analyses), reflecting the frequent development of acquired resistance [9–11]. Common treatment-related toxicities include hyperphosphatemia, central serous retinopathy, and other ocular events, often requiring dose modifications or treatment interruptions [11, 12].

Although *FGFR3* alterations are common in early-stage bladder cancer, particularly in NMIBC, a distinct subset of MIBC displays *FGFR1* amplification or overexpression [4, 13, 14]. Elevated FGFR1 levels have been linked to poor prognosis and increased tumor proliferation through MAPK pathway activation [13, 15]. Molecular profiling has identified a stromal-rich MIBC subtype characterized by high FGFR1 and low expression of luminal (KRT20) and basal (KRT5) markers, consistent with a dedifferentiated phenotype that is highly resistant to cisplatin-based neoadjuvant chemotherapy [14]. Additionally, a shift from EGFR to FGFR1 signaling during sarcomatoid dedifferentiation—linked to epithelial-mesenchymal transition (EMT)—further implicates FGFR1 in promoting invasive behavior [16]. These findings suggest that FGFR1 contributes to chemoresistance and disease progression in MIBC, underscoring its potential as a therapeutic target.

Beyond FGFR signaling, the hepatocyte growth factor (HGF)-MET axis also contributes to MIBC progression. Elevated HGF levels in MIBC patients correlate with poor survival [17, 18]. Tumor-infiltrating neutrophils secrete HGF, which activates MET signaling in bladder cancer cells, promoting proliferation, invasion, and EMT [19–21]. However, the interaction between FGFR and HGF-MET signaling in bladder cancer progression remains largely unexplored.

To investigate the mechanisms underlying acquired resistance to FGFR1 inhibition in MIBC, we primarily utilized JMSU1 cells, a model of *FGFR1*-amplified MIBC, along with two erdafitinib-resistant derivatives: JMSU1-RS, generated by prolonged exposure to erdafitinib, and JMSU1-HGF-RS, established through combined treatment with erdafitinib and HGF. We demonstrate that while erdafitinib suppresses tumor growth in JMSU1 cells, prolonged exposure induces resistance, resulting in the emergence of JMSU1-RS cells. MET activation via gene amplification was identified as a key resistance mechanism. Furthermore, exogenous HGF treatment not only induces erdafitinib resistance but also accelerates the selection of *MET*-amplified populations under erdafitinib treatment, leading to sustained, HGF-independent resistance and the establishment of the JMSU1-HGF-RS subline. Both resistant derivatives exhibit marked activation of ERK signaling. Mechanistically, Src homology 2 domain-containing phosphatase 2 (SHP2) mediates FGFR1-driven ERK signaling in parental JMSU1 cells, whereas MET activation in resistant cells amplifies SHP2-ERK signaling via Grb2-associated binder 1 (GAB1), reinforcing resistance. Notably, dual FGFR1 and MET inhibition overcomes resistance, supporting combination therapy for *FGFR1*-driven MIBC.

## Results

### Erdafitinib suppresses tumor growth in an *FGFR1*-amplified bladder cancer model but induces resistance with prolonged exposure

**Fig. 1:**
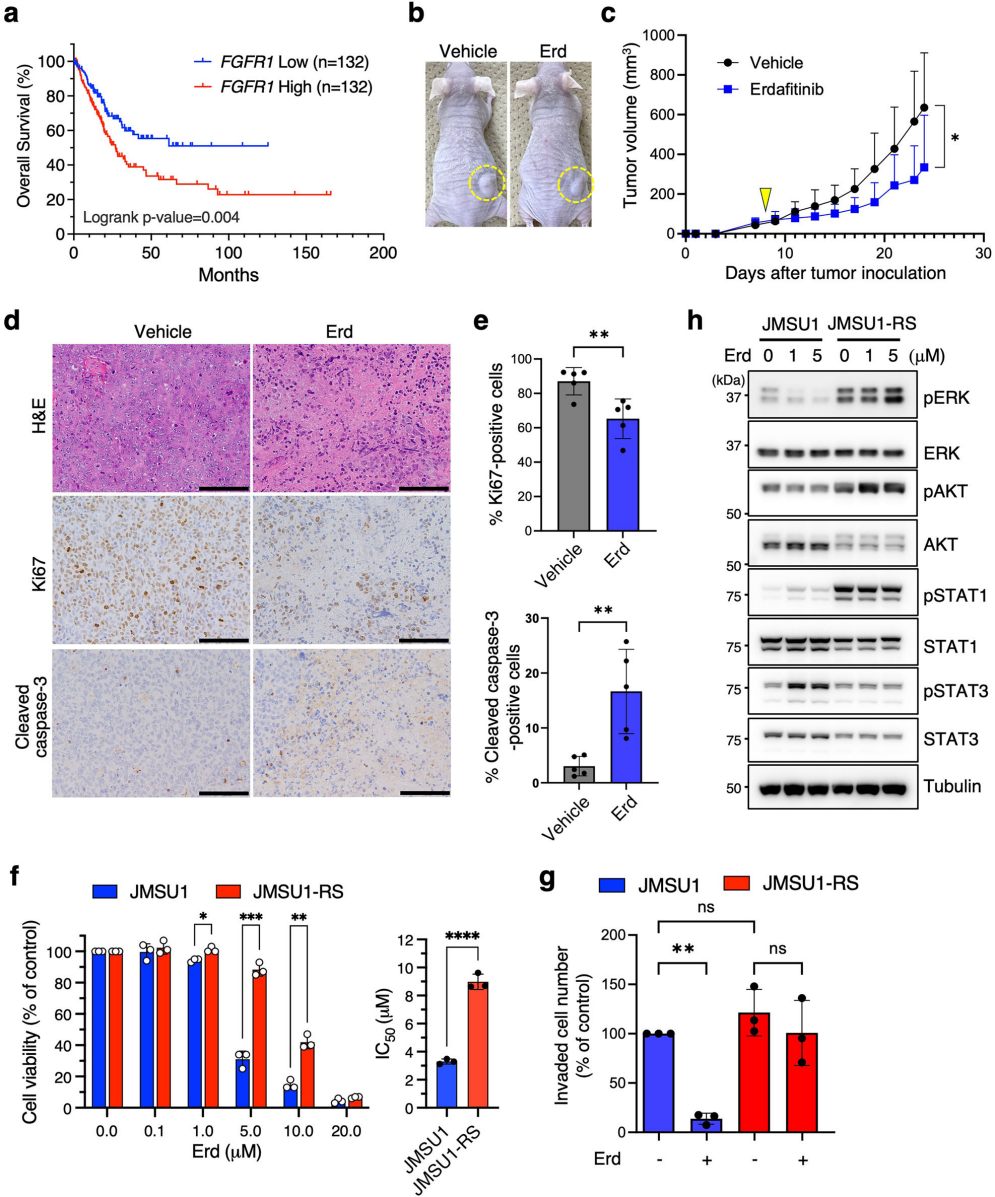
Erdafitinib suppresses tumor growth in FGFR1-amplified bladder cancer but induces resistance with prolonged exposure. **a** Kaplan-Meier survival analysis of MIBC patients with high and low *FGFR1* expression from TCGA datasets. **b** Representative images of JMSU1 xenograft tumors in mice on day 24 after tumor inoculation, with erdafitinib (Erd) or vehicle treatment initiated on day 8. **c** Tumor growth curves of the xenografts (5 mice per group). Arrowhead indicates the initiation of drug treatment (day 8). **d** Representative images of H&E, Ki67, and cleaved caspase-3-stained tumor sections collected on day 24. Scale bars, 100 µm. **e** Quantification of cells positive for Ki67 and cleaved caspase-3 (5 mice per group). **f** Viability of JMSU1 and JMSU1-RS cells treated with the indicated concentrations of Erd (left), and corresponding IC₅₀ values (right) (*n* = 3). **g** Transwell invasion assay of JMSU1 and JMSU1-RS cells treated with 1 µM Erd (+) or vehicle (−) (*n* = 3). **h** Western blot analysis of JMSU1 and JMSU1-RS cells treated with the indicated concentrations of Erd for 12 h. Data are presented as mean ± SD; *p < 0.05, **p < 0.01, ***p < 0.001, ****p < 0.0001, ns, not significant; statistical significance determined by unpaired t-test (**c**, **e**, **f**) and Tukey’s test (**g**).

Analysis of The Cancer Genome Atlas (TCGA) dataset revealed that high *FGFR1* mRNA expression correlates with poor overall survival in MIBC (Fig. 1a), supporting FGFR1 as a potential therapeutic target. To investigate its role, we evaluated the efficacy of the FGFR1 inhibitor erdafitinib in *FGFR1*-amplified JMSU1 cell line [22], which also exhibits a strong EMT signature [13, 16]. We implanted JMSU1 cells into nude mice to allow tumor formation and treated them with either vehicle or erdafitinib. Erdafitinib significantly reduced tumor growth (Fig. 1b, c). Immunohistochemistry showed that erdafitinib treatment decreased Ki67 positivity and increased cleaved caspase-3 positivity (Fig. 1d, e), indicating reduced tumor cell proliferation and increased apoptosis, consistent with effective tumor suppression.

To examine whether JMSU1 cells could develop resistance, we subjected them to high-dose erdafitinib treatment in vitro for nine weeks, generating a resistant subline, JMSU1-RS. Cell viability assays showed that JMSU1-RS cells had markedly reduced sensitivity to erdafitinib, with a mean IC₅₀ of 9.0 μM compared with 3.3 μM in parental cells (Fig. 1f). Consistently, apoptosis assays demonstrated that 5 μM erdafitinib induced a marked increase in annexin V positivity in parental cells, whereas JMSU1-RS cells showed no such response (Fig. S1), indicating resistance to erdafitinib-induced apoptosis. Furthermore, unlike parental JMSU1 cells, which lost invasive capacity following 1 μM erdafitinib treatment, JMSU1-RS cells remained invasive despite treatment (Fig. 1g), suggesting resistance preserves metastatic potential.

To explore signaling alterations associated with resistance, we examined phosphorylation levels of key FGFR1 downstream effectors. In parental cells, erdafitinib treatment (1 or 5 μM) markedly reduced phospho-ERK (pERK) levels, while pAKT showed only a modest decrease and pSTAT1/pSTAT3 exhibited slight increases (Fig. 1h). In contrast, JMSU1-RS cells displayed elevated basal pERK, pAKT, and pSTAT1, all of which remained high even after 5 μM erdafitinib treatment, indicating that resistance is associated with sustained ERK, AKT, and STAT1 activation. We also observed the reduction of total AKT, STAT1, and STAT3 levels in JMSU1-RS cells compared with parental JMSU1 cells (Fig. 1h).

### *MET* gene amplification drives acquired erdafitinib resistance

**Fig. 2:**
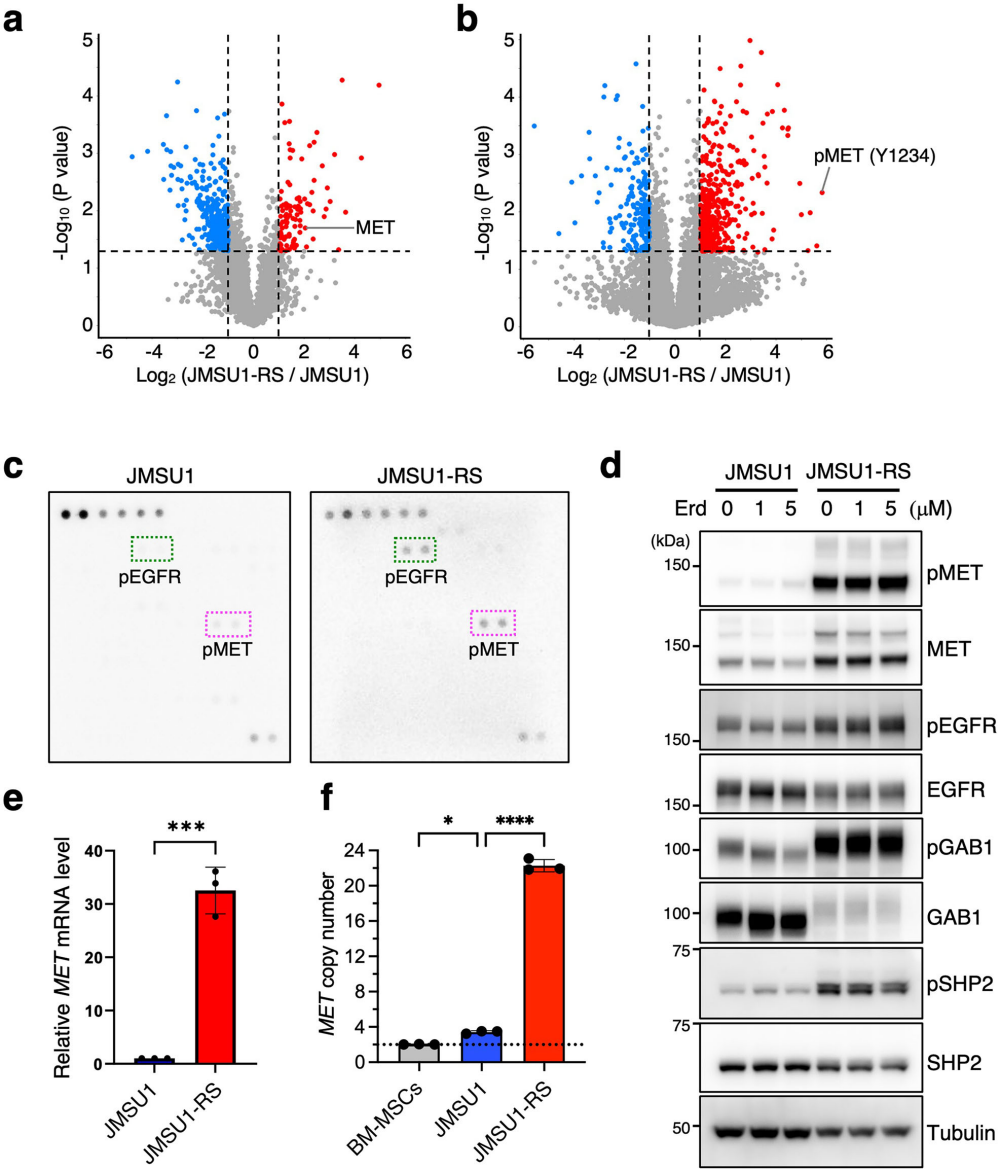
MET amplification drives acquired erdafitinib resistance in JMSU1 cells. Volcano plots depicting differentially abundant phospho-peptides (**a**) and proteins (**b**) in JMSU1-RS cells compared to JMSU1 cells. Red and blue dots indicate significantly upregulated and downregulated proteins/phospho-peptides in JMSU1-RS cells, respectively, with a fold change > 2 and *p* < 0.05. **c** Phospho-RTK array showing upregulated pMET and pEGFR in JMSU1-RS cells. **d** Western blot analysis of JMSU1 and JMSU1-RS cells treated with the indicated concentrations of erdafitinib (Erd) for 12 h. **e** Quantitative RT-PCR analysis showing *MET* mRNA upregulation in JMSU1-RS cells (*n* = 3). **f** Digital PCR analysis of *MET* gene copy number in JMSU1 and JMSU1-RS cells (*n* = 3). The dotted line represents the normal diploid copy number (two copies), as observed in BM-MSCs. Data are presented as mean ± SD; *p < 0.05, ***p < 0.001, ****p < 0.0001; statistical significance determined by unpaired t-test (**e**) and Tukey’s test (**f**).

To understand the molecular basis of resistance, we performed proteome analyses. Notably, phospho-proteome analysis identified MET, phosphorylated at Tyr1234, a site essential for kinase activity, as the most enriched phosphorylated protein in JMSU1-RS cells (Fig. 2a). Furthermore, total proteome analysis identified MET as an enriched protein in JMSU1-RS cells (Fig. 2b). A phospho-receptor tyrosine kinase (RTK) array also revealed elevated pEGFR alongside pMET in JMSU1-RS cells (Fig. 2c). Western blot analysis confirmed elevated total MET, pMET, and pEGFR levels in JMSU1-RS cells, while total EGFR remained unchanged (Fig. 2d). Notably, pMET and pEGFR levels remained elevated despite erdafitinib treatment (Fig. 2d). In addition, reduced expression of GAB1 and SHP2 was observed in JMSU1-RS cells (Fig. 2d).

To validate these findings in an independent model, we used J82 cells, which are highly invasive and express high levels of FGFR1 [21, 23], along with an erdafitinib-resistant derivative (J82-RS) generated through prolonged drug exposure. J82-RS cells exhibited markedly reduced sensitivity to erdafitinib, with a significantly higher mean IC₅₀ in viability assays (42.6 μM vs. 8.7 μM in parental J82 cells; Fig. S2a). Consistently, apoptosis assays demonstrated that 10 μM erdafitinib strongly increased annexin V positivity in parental J82 cells, whereas this effect was significantly attenuated in J82-RS cells (Fig. S2b). Moreover, J82-RS cells exhibited elevated pMET levels compared with parental cells (Fig. S2c).

To examine the underlying mechanism of MET overexpression, we performed quantitative RT-PCR analysis, which revealed a significant upregulation of *MET* mRNA in JMSU1-RS cells (Fig. 2e). Digital PCR further demonstrated a substantial increase in *MET* gene copy number, rising from an average of 3.4 copies in parental JMSU1 cells to 22.3 copies in JMSU1-RS cells (Fig. 2f). This substantial amplification likely accounts for the elevated *MET* mRNA and protein expression observed in JMSU1-RS cells. Importantly, *HGF* mRNA levels were almost negligible in both parental and resistant cells compared to the positive control KG-1 cells (Fig. S3), suggesting that endogenous *HGF* expression is unlikely to contribute to erdafitinib resistance in this model.

### MET activation sustains oncogenic signaling and promotes erdafitinib resistance

**Fig. 3:**
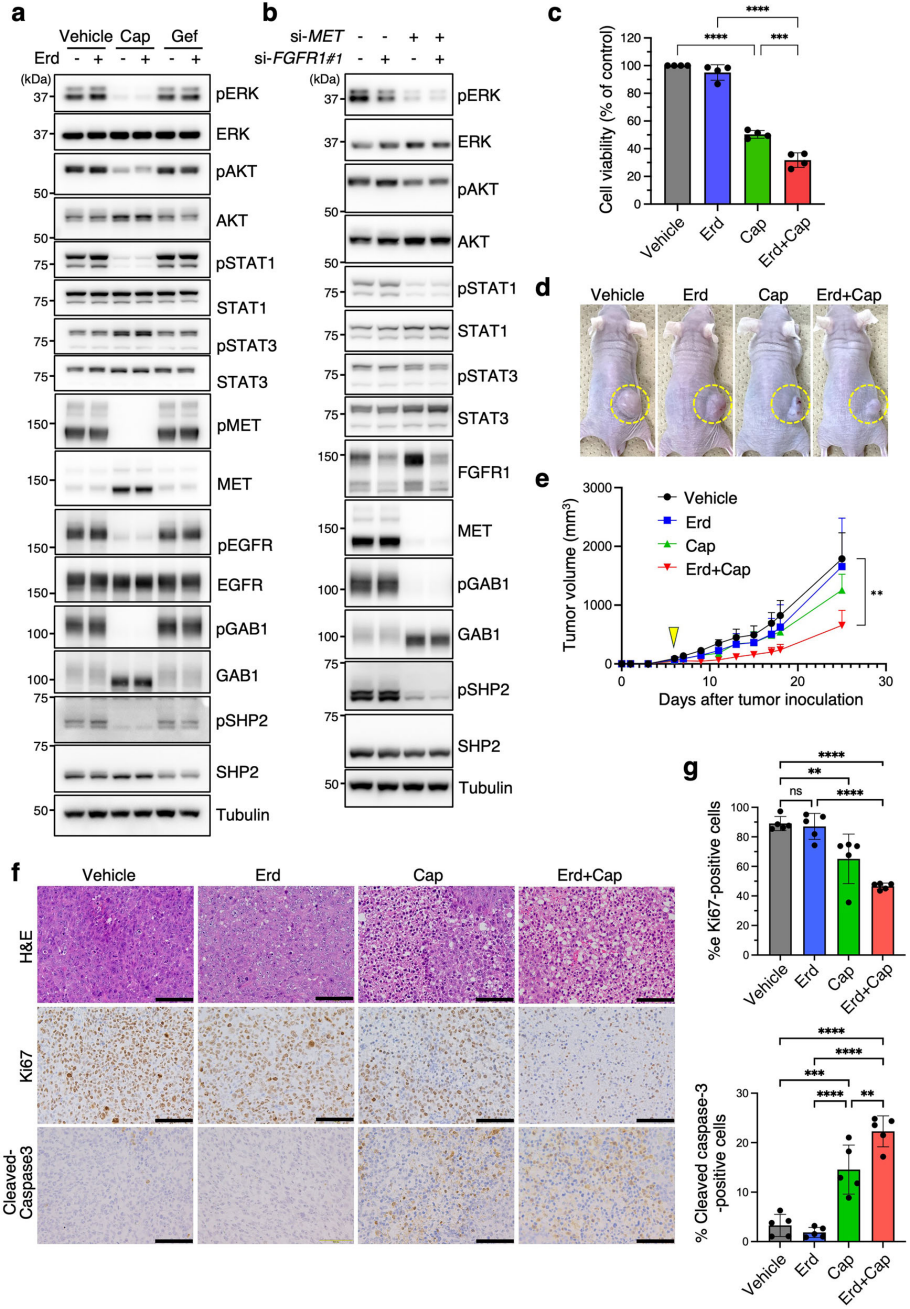
MET activation sustains oncogenic signaling and promotes erdafitinib resistance in JMSU1-RS cells. **a**, **b** Western blot analysis of JMSU1-RS cells treated with the indicated drugs (**a**) or siRNAs (**b**). Erd; erdafitinib (1 µM); Cap, capmatinib (1 µM); Gef, gefitinib (1 µM). **c** Viability of JMSU1-RS cells treated with Erd (1 µM) or Cap (1 µM) alone or in combination (*n* = 4). **d** Representative images of JMSU1-RS xenograft tumors in mice on day 25 after tumor inoculation, with drug treatment (vehicle, Erd, Cap, or Erd+Cap) initiated on day 6. **e** Tumor growth curves of the xenografts (5 mice per group). Arrowhead indicates the initiation of drug treatment (day 6). **f** Representative images of H&E, Ki67, and cleaved caspase-3-stained tumor sections collected on day 25. Scale bars, 100 µm. **g** Quantification of cells positive for Ki67 and cleaved caspase-3 (5 mice per group). Data are presented as mean ± SD; **p < 0.01, ***p < 0.001, ****p < 0.0001, ns, not significant; statistical significance determined by Tukey’s test (**c**, **e**, **g**).

To assess whether MET activation drives resistance, we treated JMSU1-RS cells with capmatinib (MET inhibitor) or gefitinib (EGFR inhibitor), alone or in combination with erdafitinib. We used 1 μM erdafitinib, a dose that did not affect JMSU1-RS cells viability (Fig. 1f), to evaluate its potential synergy with other inhibitors. Capmatinib, but not gefitinib, significantly reduced pERK, pAKT, and pSTAT1 levels (Fig. 3a), confirming that MET signaling predominates in erdafitinib resistance. Capmatinib also reduced pEGFR levels (Fig. 3a), suggesting that EGFR phosphorylation is MET-dependent. This aligns with previous findings that the MET inhibitor PHA-665752 reduces pEGFR levels, while gefitinib has minimal impact in *MET*-amplified lung cancer cells [24]. siRNA-mediated MET knockdown reduced pERK, pAKT, and pSTAT1 levels, whereas FGFR1 knockdown had minimal impact (Fig. 3b), consistent with the responses observed with capmatinib and erdafitinib, though possibly influenced by incomplete FGFR1 knockdown efficiency.

Functional assays revealed that combination of capmatinib and erdafitinib produced the strongest reduction in cell viability compared with either agent alone in JMSU1-RS and J82-RS cells, suggesting a synergistic effect (Fig. 3c and Fig. S2d). Whereas phosphorylation assays provided a sharp on/off readout of target inhibition, the viability assay was more sensitive to incremental changes, thereby revealing the synergistic effect of dual inhibition. In contrast, capmatinib did not inhibit invasion of JMSU1-RS cells and even appeared to enhance it, and the combination treatment led to only a slight reduction compared to vehicle control (Fig. S4a), suggesting that the increased invasiveness associated with acquired erdafitinib resistance may involve a MET-independent mechanism.

In mouse xenograft models, monotherapy with either erdafitinib or capmatinib had minimal impact on tumor growth, whereas combination treatment significantly reduced tumor volume (Fig. 3d, e), highlighting the efficacy of dual inhibition in overcoming resistance. This was further supported by Ki67 staining, which confirmed that the combination exerted the strongest anti-proliferative effect (Fig. 3f, g). In contrast, cleaved caspase-3 positivity was markedly increased by capmatinib alone, whereas erdafitinib had little effect either alone or in combination (Fig. 3f, g), indicating that capmatinib exerts the dominant pro-apoptotic effect in JMSU1-RS xenografts in vivo.

### HGF stimulation induces erdafitinib resistance in parental JMSU1 cells and accelerates the development of *MET*-amplified subpopulations with ligand-independent resistance

**Fig. 4:**
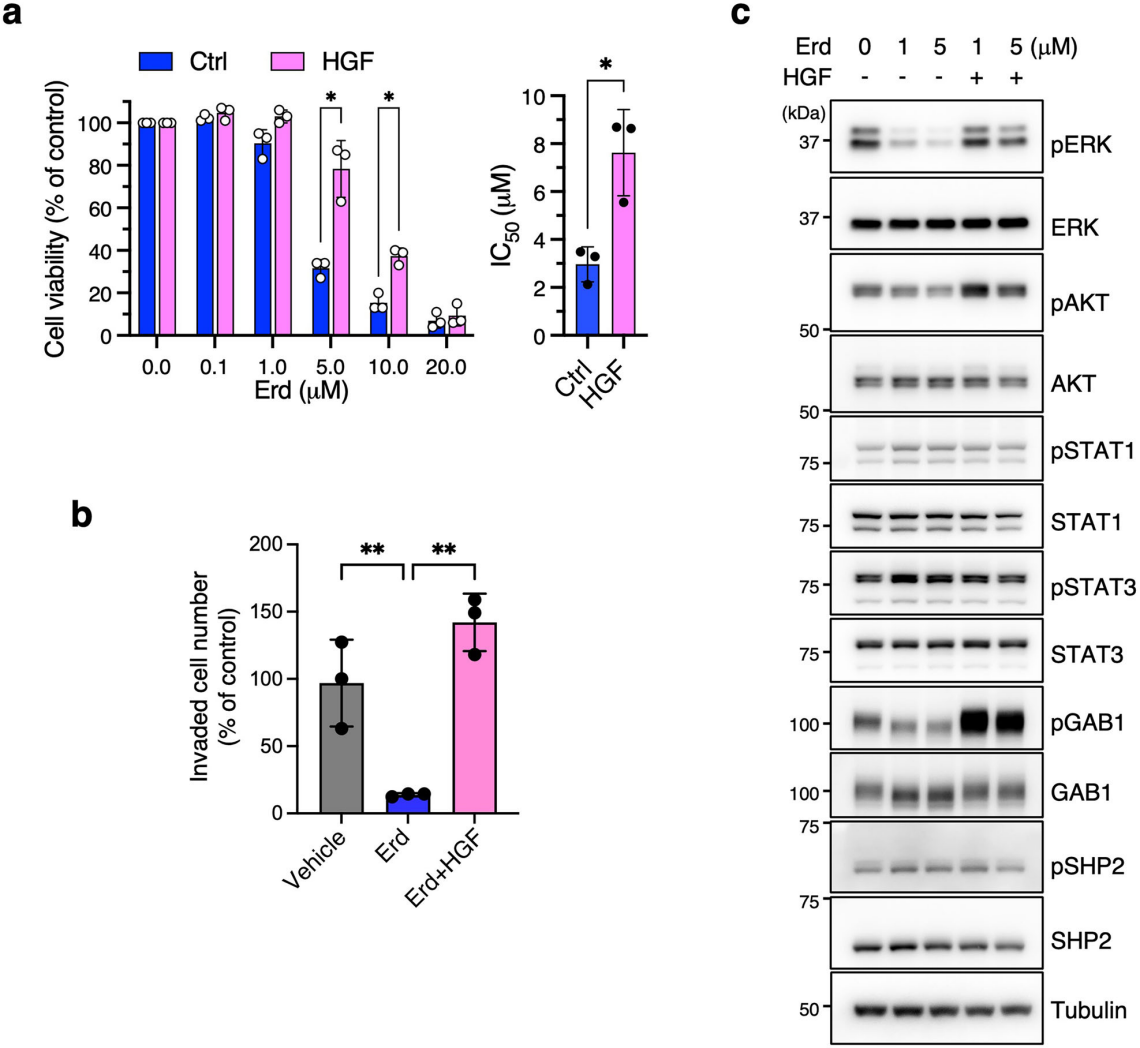
HGF stimulation induces erdafitinib resistance in parental JMSU1 cells. **a** Viability of JMSU1 cells treated with the indicated concentrations of erdafitinib (Ed) with or without 50 ng/ml HGF (left), and corresponding IC₅₀ values (right). (*n* = 3). **b** Transwell invasion assay of JMSU1 cells treated with Erd (1 µM) alone or with 50 ng/ml HGF (*n* = 3). **c** Western blot analysis of JMSU1 cells treated with Erd (1 µM or 5 µM) alone or with HGF (50 ng/ml). Data are presented as mean ± SD; *p < 0.05, **p < 0.01; statistical significance determined by unpaired t-test (**a**) and Tukey’s test (**b**).

We next investigated the effect of the MET ligand HGF on erdafitinib sensitivity in JMSU1 cells. To this end, cells were treated with erdafitinib alone or in combination with HGF. Notably, HGF counteracted the effect of erdafitinib on cell viability (Fig. 4a), apoptosis as indicated by annexin V positivity (Fig. S5), and invasive capacity (Fig. 4b), thereby inducing resistance to erdafitinib. Western blot analysis showed that erdafitinib reduced pERK levels under control conditions, but this effect was abolished in the presence of HGF (Fig. 4c), indicating that HGF sustains ERK activation despite FGFR inhibition. Similarly, HGF reversed the modest reduction in pAKT levels and further increased them beyond those in untreated controls, mimicking the signaling profile of JMSU1-RS cells (Fig. 4c). In contrast, pSTAT1 and pSTAT3 levels remained unchanged (Fig. 4c), suggesting that HGF-mediated resistance is primarily driven by ERK and AKT activation.

**Fig. 5:**
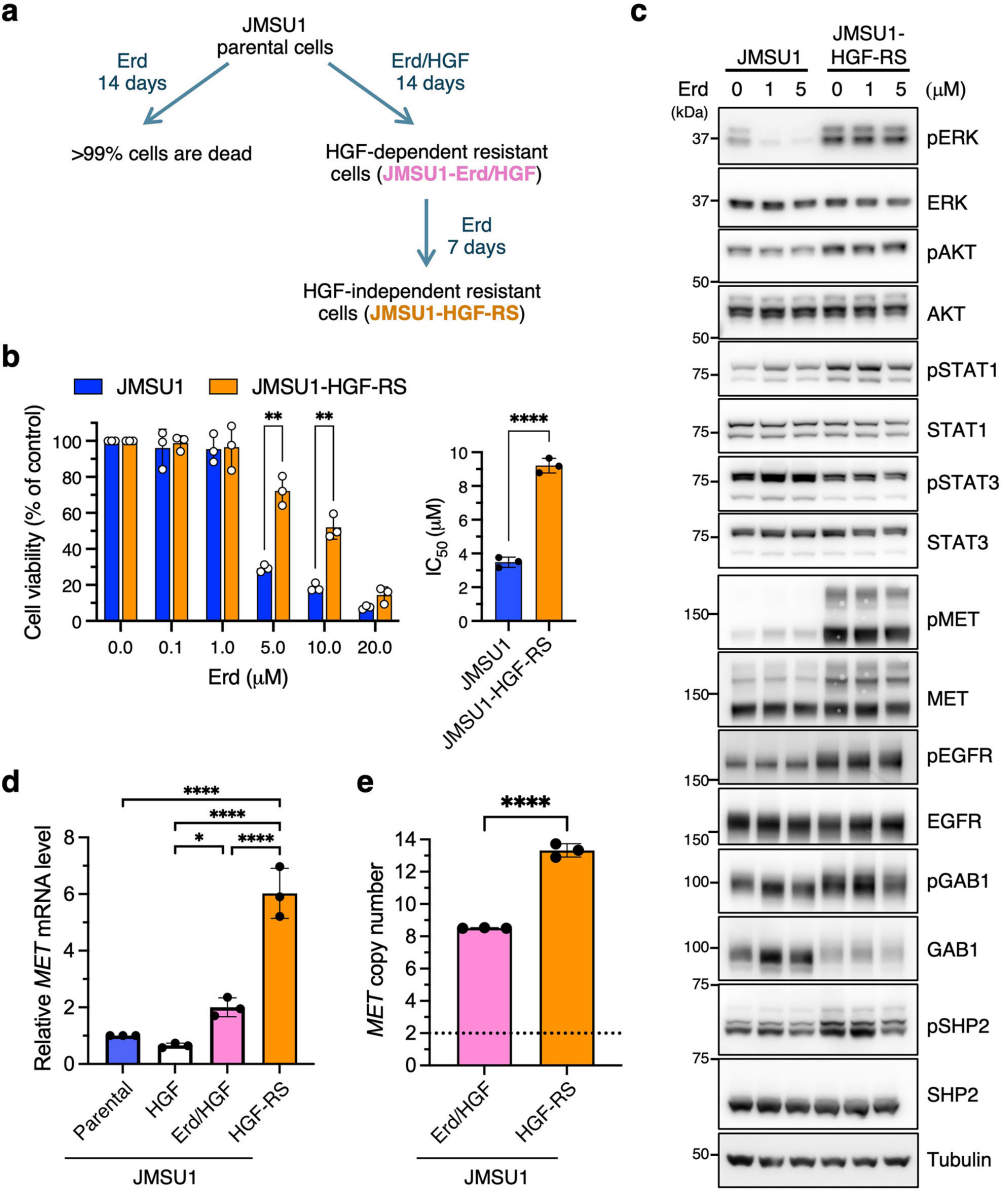
Simultaneous HGF and erdafitinib exposure promotes MET amplification and ligand-independent resistance. **a** Schematic representation of JMSU1-Erd/HGF and JMSU1-HGF-RS cell generation. **b** Viability of JMSU1 and JMSU1-HGF-RS cells treated with the indicated concentrations of erdafitinib (Erd) (left), and corresponding IC₅₀ values (right) (*n* = 3). **c** Western blot analysis of JMSU1 and JMSU1-HGF-RS cells treated with erdafitinib. **d** Quantitative RT-PCR analysis showing *MET* mRNA expression in parental JMSU1, JMSU1-Erd/HGF, and JMSU1-HGF-RS cells (*n* = 3). JMSU1 cells treated with HGF alone for 14 days (JMSU1-HGF) was also included. **e** Digital PCR analysis of *MET* gene copy number in JMSU1-Erd/HGF and JMSU1-HGF-RS cells (*n* = 3). The dotted line represents the normal diploid copy number (two copies). Data are presented as mean ± SD; *p < 0.05, **p < 0.01, ****p < 0.0001; statistical significance determined by unpaired t-test (**b, e**) and Tukey’s test (**d**).

Co-treatment with HGF and erdafitinib enabled a subset of JMSU1 cells to survive for at least 14 days, a time point at which over 99% of cells were nonviable under erdafitinib monotherapy. These surviving cells were designated JMSU1-Erd/HGF cells (Fig. 5a). Notably, a fraction of this population continued to proliferate in the presence of erdafitinib even after HGF withdrawal, giving rise to a ligand-independent, resistant subline termed JMSU1-HGF-RS cells (Fig. 5a). Like JMSU1-RS cells, JMSU1-HGF-RS cells exhibited erdafitinib resistance, as evidenced by sustained viability with a significantly higher mean IC₅₀ (9.2 μM; Fig. 5b), lack of apoptosis induction (Fig. S1), and preserved invasive capacity (Fig. S4b). Western blot analysis confirmed persistent ERK and MET activation, along with increased MET protein expression (Fig. 5c), mirroring the signaling profile of JMSU1-RS cells. In addition, reduced GAB1 expression was observed in JMSU1-HGF-RS cells (Fig. 5c).

Quantitative RT-PCR showed a significant increase in *MET* mRNA levels in JMSU1-HGF-RS cells (Fig. 5d). In contrast, HGF treatment alone for 14 days did not elevate *MET* mRNA expression (Fig. 5d), indicating that erdafitinib-driven selective pressure is essential for *MET* upregulation. Notably, *MET* gene copy number increased from 3.4 in parental JMSU1 cells (Fig. 2f) to 8.5 in JMSU1-Erd/HGF cells and 13.3 in JMSU1-HGF-RS cells (Fig. 5e). These findings suggest that concurrent exposure to HGF and erdafitinib promotes the selection of *MET*-amplified resistant subpopulations, ultimately driving stable, ligand-independent resistance.

### Dual MET and FGFR1 inhibition overcomes resistance in JMSU1-HGF-RS cells

**Fig. 6:**
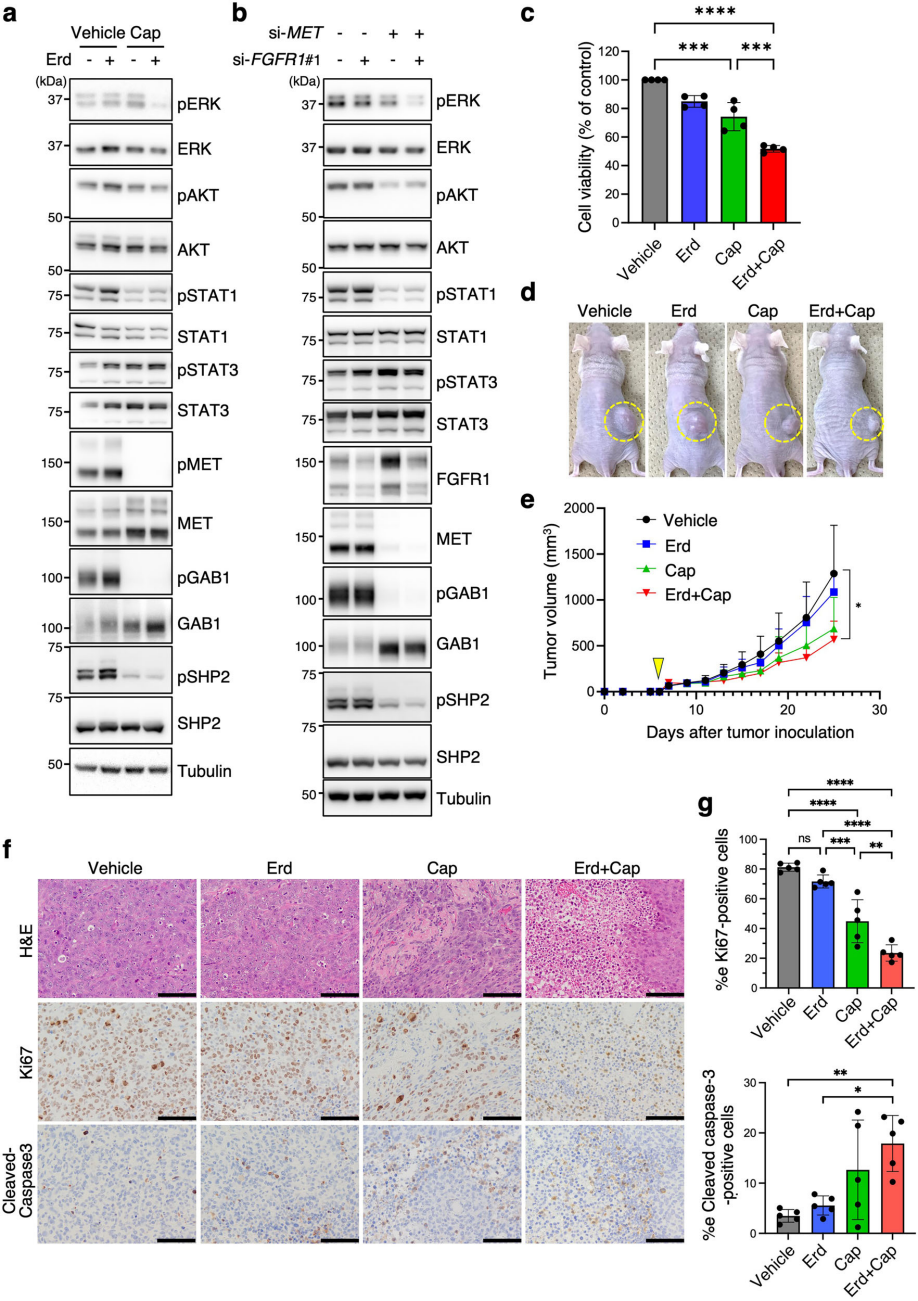
Dual MET and FGFR1 inhibition overcomes resistance in JMSU1-HGF-RS cells. **a**, **b** Western blot analysis of JMSU1-HGF-RS cells treated with indicated drugs (**a**) or siRNAs (**b**). Erd; erdafitinib (1 µM); Cap, capmatinib (1 µM). **c** Viability of JMSU1-HGF-RS cells treated with Erd (1 µM) or Cap (1 µM) alone or in combination (*n* = 4). **d** Representative images of JMSU1-HGF-RS xenograft tumors in mice on day 25 after tumor inoculation, with drug treatment (vehicle, Erd, Cap, or Erd+Cap) initiated on day 6. **e** Tumor growth curves of the xenografts (5 mice per group). Arrowhead indicates the initiation of drug treatment (day 6). **f** Representative images of H&E, Ki67, and cleaved caspase-3-stained tumor sections collected on day 25. Scale bars, 100 µm. **g** Quantification of cells positive for Ki67 and cleaved caspase-3 (5 mice per group). Data are presented as mean ± SD; *p < 0.05, **p < 0.01, ***p < 0.001, ****p < 0.0001; statistical significance determined by Tukey’s test (**c**, **e**, **g**).

Unlike JMSU1-RS cells, capmatinib alone did not reduce pERK levels in JMSU1-HGF-RS cells, but co-treatment with erdafitinib suppressed pERK signaling (Fig. 6a). This difference likely reflects distinct signaling dependencies of the two resistant models: JMSU1-RS cells are primarily MET-driven, whereas JMSU1-HGF-RS cells rely on both FGFR1 and MET for ERK activation. Similarly, simultaneous knockdown of MET and FGFR1 was required to suppress pERK levels (Fig. 6b), indicating that dual inhibition is necessary for complete ERK pathway suppression, although this may be partly influenced by incomplete FGFR1 knockdown efficiency. Consistent with these findings, capmatinib alone had a modest effect on viability, whereas its combination with erdafitinib significantly reduced viability (Fig. 6c). However, neither treatment significantly inhibited invasion (Fig. S4b). In xenograft models, JMSU1-HGF-RS tumors showed minimal response to monotherapy, while combination treatment significantly reduced tumor volume (Fig. 6d, e). Ki67 and cleaved caspase-3 staining further confirmed that dual inhibition exerted the strongest anti-proliferative and pro-apoptotic effects (Fig. 6f, g). However, although cleaved caspase-3 levels increased upon dual treatment, the relatively large deviations in the quantification suggest that the observed reduction in tumor volume may be driven primarily by anti-proliferative rather than pro-apoptotic effects.

### GAB1-SHP2 signaling mediates MET-driven erdafitinib resistance

Our findings indicate that FGFR1 and MET primarily drive ERK activation in parental and resistant JMSU1 cells, respectively. To further investigate the mechanisms underlying ERK activation in erdafitinib resistance, we examined the role of GAB1 and SHP2, key mediators of RTK signaling, including FGFR and MET pathways [25]. GAB1 functions as a scaffolding adaptor that, upon phosphorylation, recruits SHP2, linking it to active RTKs. This interaction facilitates SHP2 phosphorylation and activation, which is crucial for sustained ERK signaling [26–28].

**Fig. 7:**
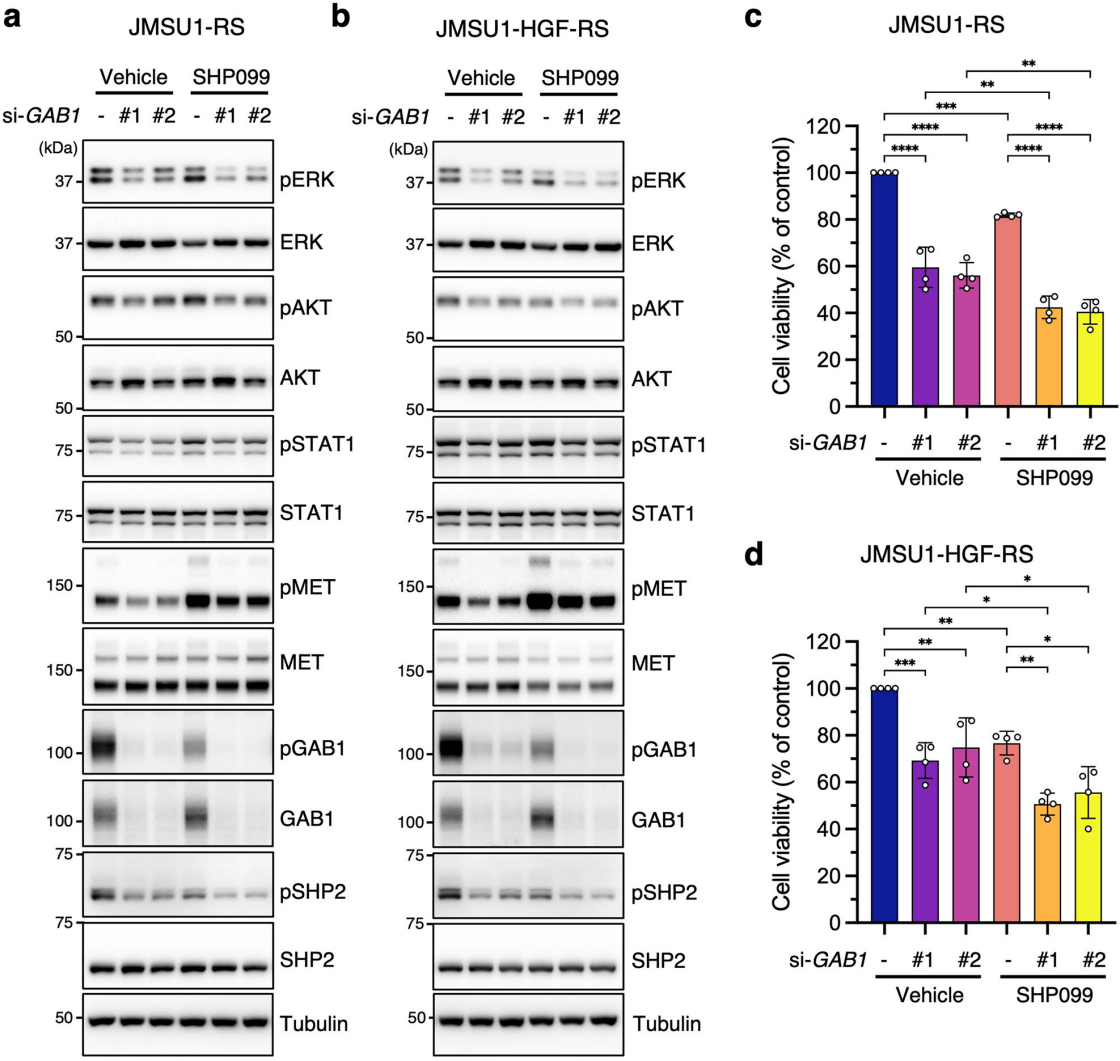
GAB1-SHP2 signaling mediates MET-driven erdafitinib resistance. Western blot analysis of JMSU1-RS (**a**) and JMSU1-HGF-RS (**b**) cells treated with *GAB1* siRNAs and SHP099. siRNA-transfected cells were treated with SHP099 (10 µM) or vehicle for 2 h before cell lysis. **c, d** Viability of JMSU1-RS (**c**) and JMSU1-HGF-RS (**d**) cells treated with *GAB1* siRNAs and SHP099. siRNA-transfected cells were treated with SHP099 (10 µM) or vehicle for 2 d, followed by a WST-8 assay (*n* = 4). Data are presented as mean ± SD; *p < 0.05, **p < 0.01, ***p < 0.001, ****p < 0.0001; statistical significance determined by Tukey’s test (**c**, **d**).

Western blot analysis showed increased pGAB1 and pSHP2 in JMSU1-RS and JMSU1-HGF-RS cells compared to parental cells (Figs. 2d, 5c). These phosphorylation signals were markedly reduced by capmatinib treatment (Figs. 3a, 6a) or MET knockdown (Figs. 3b, 6b), confirming that their activation is MET-dependent. GAB1 knockdown reduced pSHP2 levels, while SHP2 inhibition (SHP099) decreased pGAB1 levels (Fig. 7a, b). Both GAB1 depletion and SHP099 treatment reduced pERK levels, with the strongest effect observed upon combined treatment (Fig. 7a, b), suggesting that GAB1 and SHP2 cooperatively regulate ERK activation in resistant cells. Unexpectedly, GAB1 knockdown also reduced pMET levels despite unchanged total MET (Fig. 7a,b), suggesting a potential role for GAB1 in sustaining MET phosphorylation.

To evaluate the functional role of GAB1–SHP2 signaling in resistance, we assessed cell viability following GAB1 knockdown and SHP2 inhibition. Both interventions significantly reduced viability in JMSU1-RS and JMSU1-HGF-RS cells, with the greatest reduction observed upon combined treatment (Fig. 7c, d). These findings indicate that simultaneous inhibition of GAB1 and SHP2 synergistically impairs the survival of erdafitinib-resistant cells.

We next investigated the effects of GAB1 knockdown and SHP2 inhibition on ERK signaling and cell viability in parental JMSU1 cells. In contrast to resistant cells, GAB1 knockdown alone did not reduce pERK levels, even when combined with SHP099, whereas SHP099 alone effectively suppressed pERK levels (Fig. S6a). Similarly, GAB1 knockdown had no effect on pSHP2, while SHP099 reduced pGAB1 levels (Fig. S6a). Consistent with these findings, SHP099 significantly reduced cell viability, while GAB1 knockdown alone had no effect (Fig. S6b). However, the combination led to a greater reduction in viability than SHP099 alone, suggesting a cooperative role of GAB1 and SHP2 in maintaining cell survival despite minimal effects on pERK and pAKT levels.

## Discussion

**Fig. 8:**
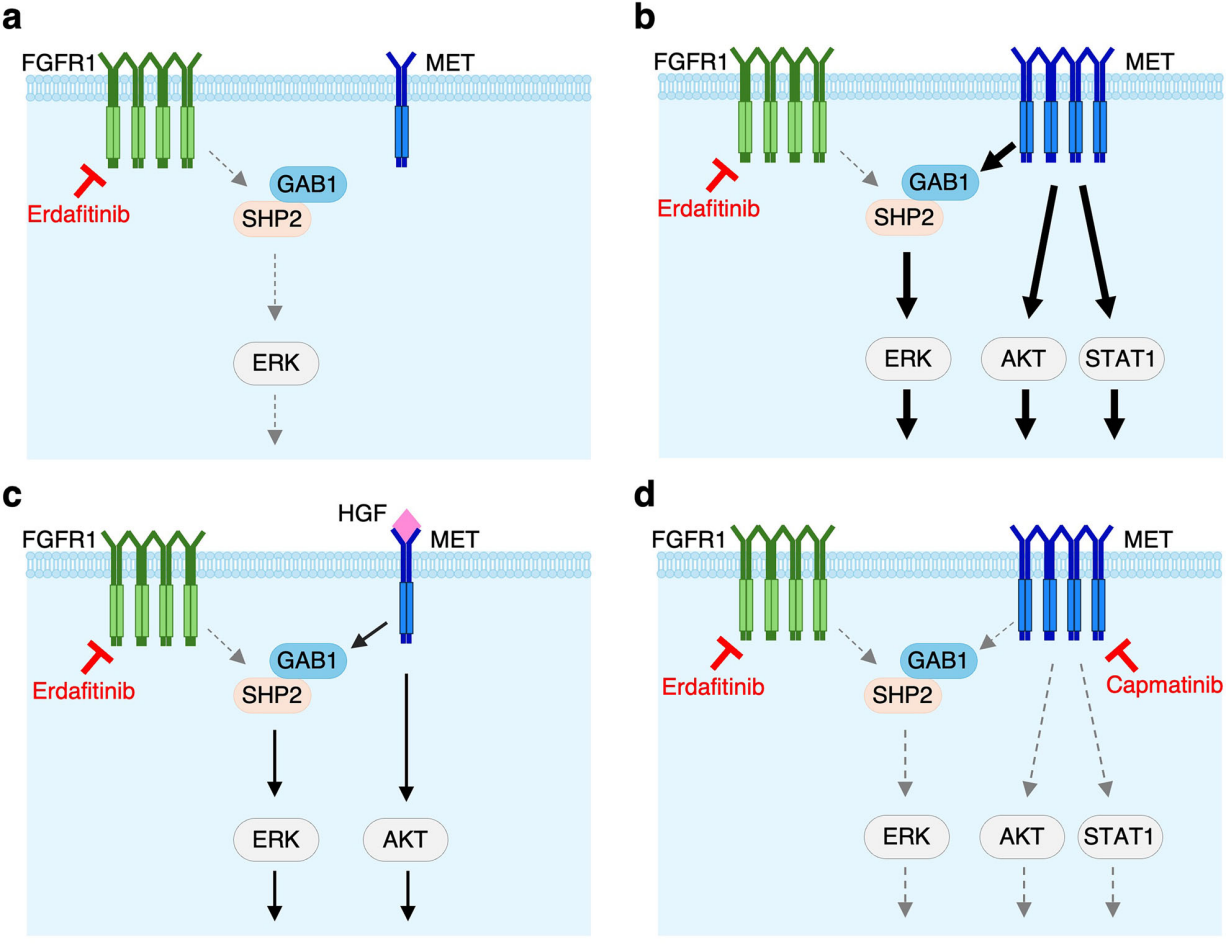
Schematic diagram of erdafitinib resistance mechanisms and therapeutic strategies. **a** Erdafitinib treatment suppresses FGFR1-mediated ERK activation, likely through SHP2 in complex with GAB1, in FGFR1-overexpressing MIBC cells. **b** Overexpression of MET predominantly activates GAB1-SHP2-ERK signaling, along with AKT and STAT1 pathways, contributing to erdafitinib resistance. **c** Exogenous HGF exposure induces erdafitinib resistance via activation of ERK and AKT signaling. **d** Dual inhibition of FGFR1 and MET overcomes erdafitinib resistance by suppressing GAB1-SHP2-ERK, AKT, and STAT1 signaling pathways.

In this study, we demonstrate that erdafitinib effectively suppresses tumor growth in JMSU1 cells, a model of *FGFR1*-amplified MIBC. Among downstream FGFR signaling effectors, activation of ERK, likely mediated through SHP2 in complex with GAB1, was most effectively suppressed by erdafitinib treatment (Fig. 8a). Interestingly, erdafitinib exposure in parental JMSU1 cells caused a paradoxical increase in pSTAT1 and pSTAT3 levels, suggesting that FGFR blockade can trigger compensatory JAK/STAT activation as a survival mechanism, consistent with previous reports of compensatory STAT3 activation upon kinase inhibition [29]. With prolonged exposure, however, cells acquired resistance, characterized by the sustained activation of ERK, AKT, and STAT1, allowing cancer cells to bypass FGFR1 inhibition (Fig. 8b). We identified MET activation via *MET* gene amplification as a primary mechanism of acquired resistance. Notably, resistant cells exhibited increased phosphorylation of GAB1 and SHP2, and their inhibition effectively suppressed ERK signaling and reduced cell viability. Given that GAB1 serves as a critical adaptor for MET pathway [30–32], it is likely that overexpressed MET predominantly activates GAB1-SHP2-ERK signaling, thereby contributing to erdafitinib resistance (Fig. 8b).

In addition, resistant cells showed elevated basal pSTAT1 but not pSTAT3 levels, and only pSTAT1 was reduced by MET inhibition, indicating that STAT1 activation is tightly linked to MET activity. By contrast, STAT3 appeared more resilient, remaining stable or only modestly increased under these conditions. These observations suggest that STAT1 primarily reflects MET-driven signaling, while STAT3 contributes more broadly to adaptive responses during FGFR inhibition. Thus, erdafitinib resistance involves both MET–GAB1–SHP2 activation and reprogramming of JAK/STAT signaling, with distinct roles for STAT1 and STAT3.

Beyond phosphorylation changes, JMSU1-RS cells also showed reduced total GAB1, AKT, STAT1, and STAT3 levels compared with parental JMSU1 cells, suggesting that resistance acquired under continuous FGFR inhibition with *MET* amplification entails broader signaling reprogramming that affects both protein abundance and phosphorylation dynamics to sustain survival under therapeutic pressure. In contrast, while JMSU1-HGF-RS cells also exhibited reduced total GAB1, their total AKT, STAT1, and STAT3 levels remained largely unchanged, indicating that resistance selected in the presence of exogenous HGF engages a distinct signaling program despite converging on *MET* amplification.

Of note, our data suggest the presence of a reciprocal regulatory relationship between FGFR1 and MET. Specifically, MET silencing increased FGFR1 expression in JMSU1-RS and JMSU1-HGF-RS cells (Figs. 3b, 6b), while FGFR1 inhibition in parental JMSU1 cells led to increased MET expression. This bidirectional regulatory loop highlights the remarkable plasticity of RTK networks in JMSU1 cells and likely contributes to their capacity to adapt under therapeutic pressure.

Importantly, *HGF* mRNA levels were negligible in both parental and resistant JMSU1 cells. However, exposure of parental cells to exogenous HGF activated ERK and AKT, restoring their survival despite FGFR1 inhibition (Fig. 8c). This observation aligns with previous findings in EGFR-driven lung adenocarcinoma, where HGF exposure induces gefitinib resistance [33]. Intriguingly, parental JMSU1 cells originally harbored an average of 3.4 copies of the *MET* gene (Fig. 2f). Following two weeks of treatment with erdafitinib and HGF, this number increased to 8.5 copies, and an additional one-week exposure to erdafitinib alone further elevated it to 13.3 copies (Fig. 5e). These findings indicate inherent cellular heterogeneity within the parental JMSU1 population, where subpopulations with higher *MET* gene copy numbers preferentially expand under erdafitinib-mediated selective pressure, a process further accelerated by HGF stimulation. This clonal selection mechanism underlies the emergence of resistance and highlights the pivotal role of tumor heterogeneity in therapeutic resistance, consistent with observations in other cancer models [34, 35].

EMT contributes to intratumoral heterogeneity [36–39]. Recent study highlights the critical role of FGFR1 signaling in EMT of MIBC [16]. Consistently, analysis of TCGA data revealed that elevated *FGFR1* levels significantly correlated with increased expression of key mesenchymal markers, including *vimentin (VIM)* and EMT-inducing transcription factors (*ZEB1* and *SNAI1*), while inversely correlating with the epithelial marker *E-cadherin (CDH1)* in MIBC (Fig. S7a-d), further reinforcing FGFR1’s association with mesenchymal phenotypes. In line with these findings, FGFR1 knockdown in JMSU1 cells markedly reduced *VIM* and increased *CDH1* expression, indicating a shift toward an epithelial phenotype (Fig. S8). By contrast, the expression levels of *ZEB1* and *SNAI1* were only marginally affected (Fig. S8), raising the possibility that alternative EMT transcription factors (e.g., *ZEB2*, *SNAI2*, *TWIST1*) may mediate the FGFR1-driven EMT phenotype in this context. These findings suggest that aberrant FGFR1 signaling may contribute to MIBC cellular heterogeneity, including variations in *MET* gene copy numbers, through EMT-driven mechanisms. Notably, bladder cancer tissues, particularly MIBC, exhibit high levels of infiltrated neutrophils, which are known to secrete HGF [19]. Moreover, TCGA data reveal a positive correlation between *FGFR1* and *HGF* mRNA levels in MIBC (Fig. S7e). This suggests that FGFR1-driven MIBC are characterized by HGF-rich tumor microenvironments, which may promote the expansion of *MET*-amplified subpopulations within heterogeneous tumors during erdafitinib treatment. The enhanced effects of combined inhibition further suggest that FGFR1 and MET provide partly independent inputs to downstream pathways, underscoring the need to block both signals for maximal therapeutic benefit. Together, these findings underscore the importance of targeting the HGF-MET axis and supports dual FGFR1 and MET inhibition as a promising therapeutic strategy to enhance the efficacy of FGFR-targeted therapies (Fig. 8d).

Given that resistance to erdafitinib in *FGFR3*-altered bladder cancer can arise through multiple mechanisms, such as increased *P4HA2* expression, which stabilizes HIF-1α and reduces reactive oxygen species levels [40], FGFR3 gatekeeper mutations, *TP53* alterations, and off-target resistance mechanisms including PI3K-mTOR activation and EGFR bypass signaling [41, 42], it is likely that similar diverse mechanisms contribute to erdafitinib resistance in FGFR1-driven bladder cancers. A limitation of this study is the restricted number of models examined. Although we complemented the JMSU1-based system with J82-RS cells as an independent FGFR1-expressing resistant model, further validation using additional FGFR1-dependent bladder cancer models, such as UMUC3, would strengthen the generalizability of our findings. In addition, analyses of post-treatment MIBC specimens from patients who develop resistance to erdafitinib will be critical to confirm the clinical relevance of MET pathway hyperactivation.

## Materials and methods

### Cell culture and treatments

The bladder cancer cell lines JMSU1 and J82 were obtained from RIKEN (Tsukuba, Japan) and ATCC (Manassas, VA, USA), respectively. The acute leukemia cell line KG-1 was obtained from JCRB Cell Bank (Osaka, Japan). These cells were cultured in RPMI 1640 medium (Nacalai Tesque, Kyoto, Japan) supplemented with 10% fetal bovine serum (FBS) and 1% penicillin/streptomycin. Primary human bone marrow-derived mesenchymal stem cells (BM-MSCs) were purchased from PromoCell GmbH (Heidelberg, Germany) and maintained in Mesenchymal Stem Cell Basal Medium 2 with Supplement Mix (PromoCell GmbH) and 1% penicillin/streptomycin. All cells were cultured at 37 °C in a humidified 5% CO₂ atmosphere.

Cells were treated with erdafitinib, capmatinib, SHP099 (Selleck Chemicals, Houston, TX, USA), or DMSO as a vehicle control. To generate erdafitinib-resistant cells, JMSU1 and J82 cells were continuously exposed to 5 µM and 10 µM erdafitinib, respectively, for nine weeks, with the drug replenished at each passage. Surviving JMSU1 and J82 cells were designated JMSU1-RS and J82-RS, respectively. JMSU1-HGF-RS cells were generated by exposing JMSU1 cells to 5 µM erdafitinib and 50 ng/mL hepatocyte growth factor (HGF) (FUJIFILM Wako, Osaka, Japan) for two weeks, followed by one week of erdafitinib alone.

Cells were transfected with siRNAs using Lipofectamine RNAiMAX (Thermo Fisher Scientific, Waltham, MA, USA) according to the manufacturer’s instructions. Briefly, 20 nM siRNAs were mixed with RNAiMAX in OPTI-MEM, incubated for 20 minutes at room temperature, and added to cells. siRNA sequences or IDs are listed in Supplementary Table 1.

### Cell viability and apoptosis assays

Cell viability was assessed using the WST-8 assay (Cell Counting Kit-8; Dojindo, Kumamoto, Japan). Cells (2500/well) were plated in 96-well plates in triplicate and cultured overnight before treatment with drug for 48 h. For siRNA-transfected cells, transfection was performed 48 h prior to replating in 96-well plates. IC_50_ values for erdafitinib were calculated using non-linear regression analysis in GraphPad Prism 10 (GraphPad Software, Boston, MA, USA). For apoptosis assays, cells were seeded in 6-well plates (2.5 × 10⁵/well) and treated with drugs for 48 h. Apoptosis was evaluated by staining with FITC-labeled Annexin V and propidium iodide (PI) using the MEBCYTO Apoptosis Kit (MBL, Tokyo, Japan) according to the manufacturer’s instructions. Stained cells were analyzed by a BD FACSCanto II flow cytometer (BD Biosciences, San Jose, CA, USA).

### Transwell invasion assay

Transwell inserts (8 µm pore size, Corning 3422, Corning, NY, USA) were coated with 50 µL of diluted Matrigel (Corning) (1:40 in serum-free RPMI 1640) and incubated at 37 °C for 4 h. The lower surface was coated with fibronectin (10 µg/mL) at 37 °C for 1 h. Cells (25,000/well) suspended in serum-free medium were seeded into the upper chamber, while the lower chamber was filled with RPMI 1640 containing 10% (v/v) FBS as a chemoattractant. C After incubation at 37 °C for 17 h, cells on the upper membrane surface were removed, and the invaded cells on the lower surface were fixed with formalin and stained with DAPI (5 µg/mL in PBS). Fluorescence microscopy (BZ-9000; KEYENCE, Osaka, Japan) was used to visualize the invaded cells, which were subsequently quantified using ImageJ software (National Institutes of Health, Bethesda, MD, USA) [43].

### Western blotting

Cells were lysed in ice-cold lysis buffer (50 mM Tris-HCl, pH7.4, 0.5% (v/v) Nonidet P-40, 150 mM NaCl, 5 mM EDTA, 50 mM NaF, 1 mM Na3VO4) containing a protease inhibitor cocktail (Nacalai Tesque). Proteins were separated by SDS-PAGE and transferred onto polyvinylidene difluoride (PVDF) membranes (Immobilon-P; EMD Millipore). Membranes were blocked in PBS containing 5% (v/v) skim milk for 1.5 h at room temperature. After washing with PBS containing 0.1% (v/v) Tween-20, membranes were incubated with primary antibodies overnight at 4 °C, followed by HRP-conjugated secondary antibodies for 1.5 h at room temperature. Signals were detected by chemiluminescence using Immobilon® (EMD Millipore) and SuperSignal West Dura (Thermo Fisher Scientific) and imaged with an AE-9300H Ez-Capture MG imaging system (ATTO, Tokyo, Japan). Quantification of bands was performed using ImageJ macro, Band/Peak Quantification Tool, and the results are provided in the Supplementary spreadsheet (Excel file). Uncropped blots are shown in Supplementary File. Antibodies used are listed in Supplementary Table 2.

### Proteome analyses

Peptide samples were prepared from JMSU1 and JMSU1-RS cells (*n* = 3), respectively, using EasyPep™ Mini MS Sample Prep Kit (Thermo Fisher Scientific) according to the manufacturer’s instructions. Protein concentrations were measured using Pierce™ BCA Protein Assay Kit (Thermo Fisher Scientific). Proteins (300 μg) from each cell lysate were reduced, alkylated, and digested with trypsin/Lys-C. The obtained peptides were acidified and desalted by a GL-Tip SDB (GL Sciences, Tokyo, Japan). A small fraction of each desalted peptides was used for total proteome analysis. The remaining peptides were used for phospho-peptides enrichment using a High-Select™ TiO_2_ Phosphopeptide Enrichment Kit (Thermo Fisher Scientific) according to the manufacturer’s protocols.

LC-MS/MS analyses were performed using a Thermo Vanquish Neo UHPLC integrated Orbitrap Exploris 240 system equipped with a nanoelectrospray source (Thermo Fisher Scientific). Each sample was loaded onto the trap column (PepMap™ Neo Trap Cartridge, Thermo Scientific, 5 mm, C18, 5 μm) linked with a separation column packed with 3 μm C18-silica particles (12.5 cm × 75 μm capillary column, Nikkyo Technos, Tokyo, Japan). The samples were separated by a 120 min linear gradient from 4 to 40% acetonitrile with 0.1% formic acid at a flow rate of 300 nL/min. The eluted peptides were analyzed using an Orbitrap Exploris 240 in a data-independent acquisition (DIA) mode. The DIA method consisted of MS1 spectra were collected in the range of 495 to 745 *m/z* at a 30,000 resolution to set normalized AGC target (%) of 200 and a maximum injection time of 45 ms. MS2 spectra were collected in the range of 500 to 740 *m/z* at a 120,000 resolution to set normalized AGC target (%) of 1000, a maximum injection time of “Auto”, and a normalized collision energy of 25%. The isolation width for MS2 was set to 4 *m/z*, an optimized window arrangement was used in Xcalibur software (v4.7, Thermo Fisher Scientific).

The DIA-MS files were analyzed using DIA-NN (v1.9.2) to derive the quantification results of proteins and phospho-peptides. Each spectral library was generated from the human protein sequence UniProt database using DIA-NN. For identification of proteins and phospho-peptides, the peptide ions in the spectral library were filtered to achieve a false discovery rate (FDR) of less than 1%. For visualizing significantly changed proteins and phospho-peptides, volcano plots were created by plotting the log_2_(fold change) against the −log_10_(*P* value) using Perseus software (version 2.1.3.0, Max Planck Institute of Biochemistry). The resulting quantification data were log_2_-transformed, and filtered to have “2” valid values in each group. Missing values were imputed with random numbers from a normal distribution (Width = 0.3 and Down-shift = 1.8).

### Phospho-receptor tyrosine Kinase (RTK) arrays

The Human RTK Phosphorylation Antibody Array Kit (ab193662; Abcam), containing nitrocellulose membranes spotted in duplicate with 71 different anti-RTK antibodies, was used according to the manufacturer’s instructions with modifications. Briefly, JMSU1 and JMSU1-RS cells were cultured in fresh medium without drug for 24 h before collection. Protein lysates (750 μg) were incubated overnight at 4 °C with the array membranes. Bound phosphorylated RTK proteins were detected using a biotin-conjugated anti-phosphotyrosine antibody (4G10® Platinum, EMD Millipore, Burlington, MA, USA), followed by HRP-conjugated streptavidin.

### RNA isolation and quantitative RT-PCR

Total RNA was extracted using Sepasol-RNA I Super G (Nacalai Tesque) and reverse-transcribed with the PrimeScript RT reagent kit (Takara, Shiga, Japan). Real-time PCR was performed on a QuantStudio 3 system (Thermo Fisher Scientific) using Fast SYBR Green Master Mix (Thermo Fisher Scientific). The amount of mRNA was normalized relative to that of *β-Actin* RNA. The sequences of the primers used are listed in Supplementary Table 3.

### Genomic DNA isolation and TaqMan® copy number assay

Genomic DNA was extracted using the Quick-gDNA MiniPrep Kit (Zymo Research, Irvine, CA, USA). Absolute *MET* copy number was determined using a TaqMan® Copy Number Assay with reagents, instruments, and software from Thermo Fisher Scientific (Waltham, MA, USA). Digital PCR was performed on the QuantStudio 3D Digital PCR System using the TaqMan® Copy Number Assay for *MET* (Assay ID: Hs02323823_cn) and the TaqMan® Copy Number Reference Assay for RNase P (Cat. No. 4403326), following the manufacturer’s instructions. Briefly, the TaqMan PCR reaction mixture was loaded onto QuantStudio 3D digital PCR chips using the QuantStudio 3D Digital PCR Chip Loader. After PCR amplification, chips were analyzed on the QuantStudio 3D Digital PCR Instrument. Absolute quantification data were obtained using QuantStudio 3D AnalysisSuite Software. The *MET* copy number was calculated using the equation: *MET* copy number = raw *MET* number / (raw *RNase P* number / 2). Genomic DNA from BM-MSCs, which have a normal diploid *MET* copy number (two copies), was included as a reference in each run.

### Animal studies

All procedures were approved by the Fukushima Medical University School of Medicine Animal Care and Use Committee. Six-week-old male nude mice (BALB/cAJcl nu/nu) were obtained from CLEA Japan (Shizuoka, Japan). Mice were anesthetized with isoflurane and subcutaneously injected with tumor cells (2.0 × 10⁶ in 50% (v/v) Matrigel in PBS) into the flank. When tumors reached 50–100 mm³ (day 8 for parental JMSU1 cells; day 6 for JMSU1-RS and JMSU1-HGF-RS cells), mice were randomized into two or four groups and treated daily by oral gavage with erdafitinib (20 mg/kg in 0.5% sodium carboxymethylcellulose [CMC-Na]) or capmatinib (10 mg/kg in 0.5% CMC-Na) alone or in combination for 14 days (*n* = 5 per group). Tumor volume was measured every 2–3 days and calculated using the formula: Tumor volume (mm³) = (length × width²)/2. Mice were euthanized if body weight decreased over 30% from the day of administration, tumor weight exceeded 10% of body weight, or they exhibited signs of intolerable suffering.

### Pathological analysis

Tumor tissues collected from mice were fixed in 10% formalin, paraffin-embedded, sectioned (4 µm), and stained with hematoxylin and eosin (H&E). Immunohistochemistry for anti-Ki67 (ab16667, Abcam, Cambridge, UK) and anti-cleaved caspase-3 (#9661, Cell Signaling Technology, MA, USA) was performed using the Histofine Simple Stain Mouse MAX-PO (R) (Nichirei, Tokyo, Japan). Ki67 and cleaved caspase-3 positivity rates were quantified using QuPath software (Version 0.5.1-x64, University of Edinburgh, UK) [44].

### The Cancer Genome Atlas (TCGA) data analysis

Previously reported mRNA expression (RNA sequencing) and clinical data from patients with MIBC [45] were downloaded on Jun 13, 2024 from The Cancer Genome Atlas (TCGA) via cBioPortal [46, 47] (http://www.cbioportal.org). Kaplan–Meier survival curves were generated using GraphPad Prism 10 to compare overall survival between patients with high (*top third*, n = 132) and low (*bottom third*, n = 132) *FGFR1* expression. Statistical significance between survival curves was assessed using the log-rank test. Correlations between mRNA expression levels were analyzed and visualized as scatter plots using cBioPortal.

### Statistical analysis

Data were analyzed using GraphPad Prism 10. Statistical significance was assessed using an unpaired t-test for two-group comparisons and one-way ANOVA with Tukey’s or Dunnett’s post hoc test for multiple groups, assuming normal data distribution. Significance was defined as *p* < 0.05. Sample sizes were not predetermined by statistical methods. Each sample size is shown in the figure or its legend. No samples or animals were excluded from analysis. Randomization and blinding were not applied.

## Supplementary information

Supplementary figs 1–8

Supplementary tables 1–3

Original Western blots

Western blot quantification

## Data Availability

Proteomics data have been deposited to the ProteomeXchange Consortium (PXD062185, http://www.proteomexchange.org/) via the jPOST partner repository (JPST003720, https://jpostdb.org/). Further information is available from the corresponding author.
