# Supplementary material for: MET signaling drives acquired resistance to erdafitinib in muscle-invasive bladder cancer cells
Source: Cell Death Dis. 2025 Nov 28;16(1):868. doi: 10.1038/s41419-025-08221-8 (PMC12663377; doi:10.1038/s41419-025-08221-8)
Supplement: Supplementary file 1 — Supplementary figs 1–8 [file 41419_2025_8221_MOESM1_ESM.pdf]

Supplementary Figure S1

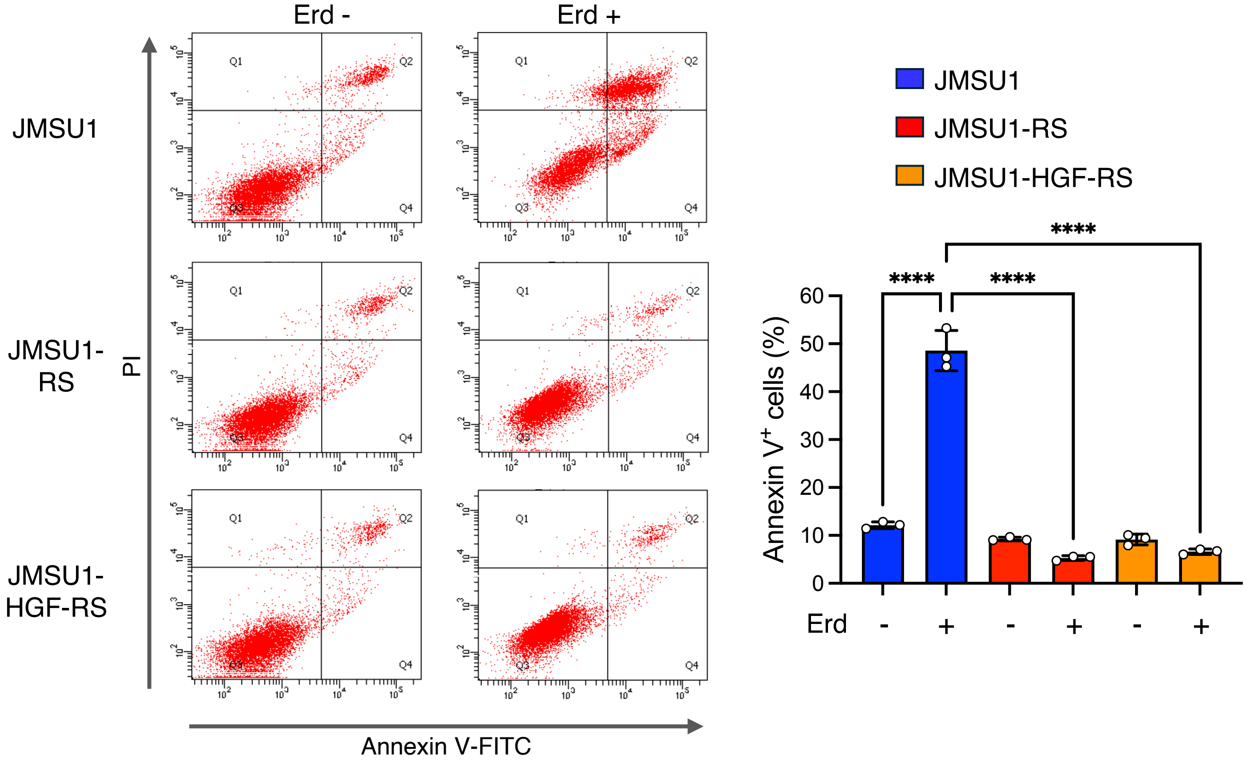

Fig. S1. Apoptosis analysis of parental JMSU1, JMSU1-RS, and JMSU1-HGF-RS cells treated with erdafitinib. Representative Annexin V/PI flow cytometry plots of cells treated with vehicle (-) or 5 µM erdafitinib (Erd) (+) for 48 h (Left). Quantification of Annexin V-positive cells (Right). Data are presented as mean ± SD (n = 3). ****p<0.0001; statistical significance determined by Tukey's test.

Supplementary Figure S2

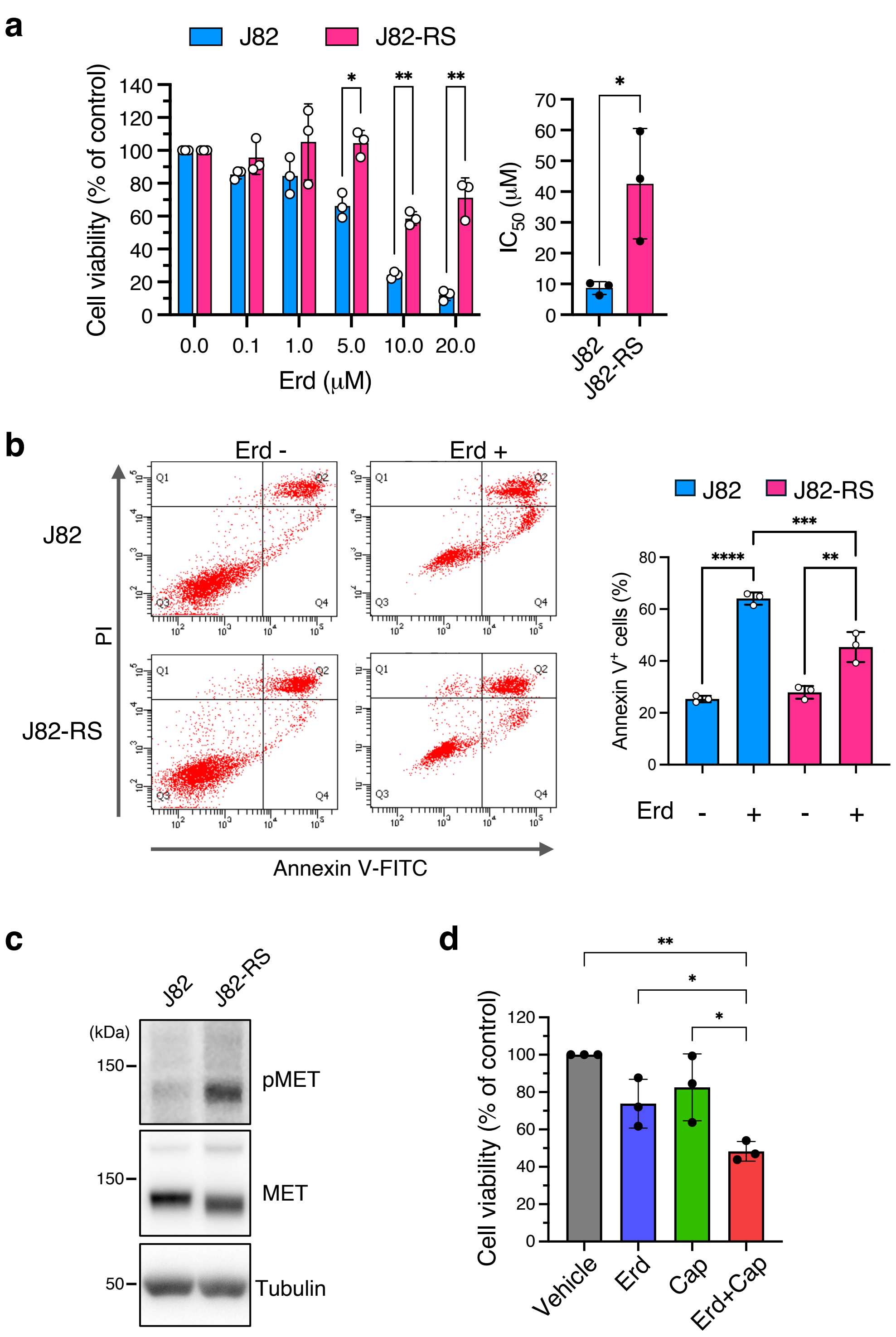

Fig. S2. Characterization of parental J82 and erdafitinib-resistant J82-RS cells. a Viability of J82 and J82-RS cells treated with the indicated concentrations of erdafitinib (Erd) (left), and corresponding IC_{50} values (right) (n = 3). **b** Representative Annexin V/PI flow cytometry plots of cells treated with vehicle (-) or 10 μM Erd (+) for 48 h (Left). Quantification of Annexin V-positive cells (Right). (n = 3). **c** Western blot analysis of J82 and J82-RS cells. **d** Viability of J82-RS cells treated with 10 μM Erd or 1 μM Capmatinib (Cap) alone or in combination (n = 3). Data are presented as mean ± SD; *p<0.05, **p<0.01, ***p<0.001, ****p<0.0001; statistical significance determined by unpaired t-test (**a**) and Tukey's test (**b, d**).

Supplementary Figure S3

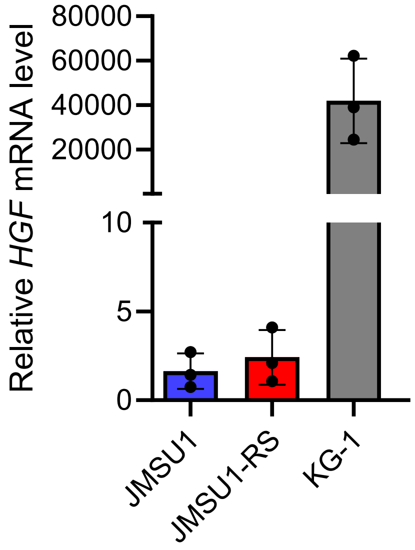

Fig. S3. *HGF* expression in parental and resistant JMSU1 cells. Quantitative RT-PCR analysis of *HGF* mRNA levels in JMSU1, JMSU1-RS, and KG-1 cells (n = 3). Data are presented as mean ± SD.

Supplementary Figure S4

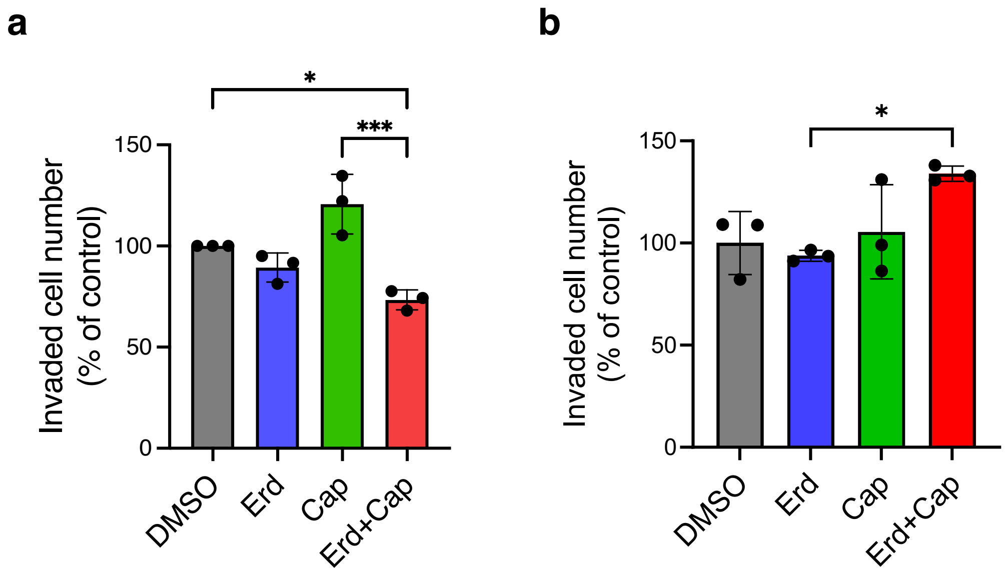

Fig. S4. Invasive capacity of resistant cells following dual inhibition. a, b Transwell invasion assays of JMSU1-RS (**a**) and JMSU1-HGF-RS (**b**) cells treated with 1 μM erdafitinib (Erd) or 1 μM capmatinib (Cap), alone or in combination (n = 3). Data are presented as mean ± SD; *p<0.05, ***p<0.001; statistical significance determined by Tukey's test.

Supplementary Figure S5

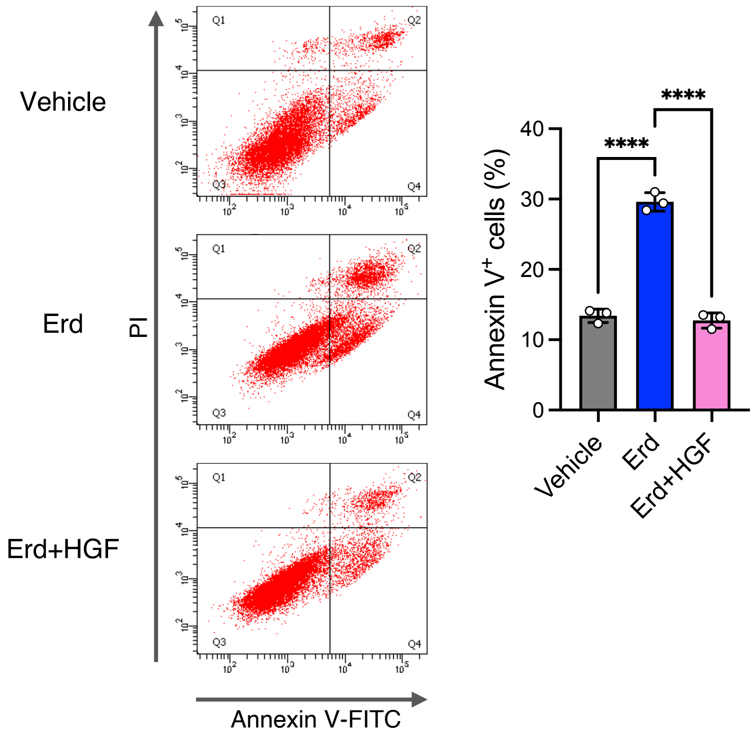

Fig. S5. HGF counteracts the pro-apoptotic effect of erdafitinib in JMSU1 cells. Representative Annexin V/PI flow cytometry plots of cells treated with vehicle, 5 µM erdafitinib (Erd), or 5 µM erdafitinib plus 50 ng/ml HGF (Erd+HGF) for 48 h (left). Quantification of Annexin V–positive cells (right). Data are presented as mean ± SD (n = 3). ****$p < 0.0001$; statistical significance determined by Tukey's test.

Supplementary Figure S6

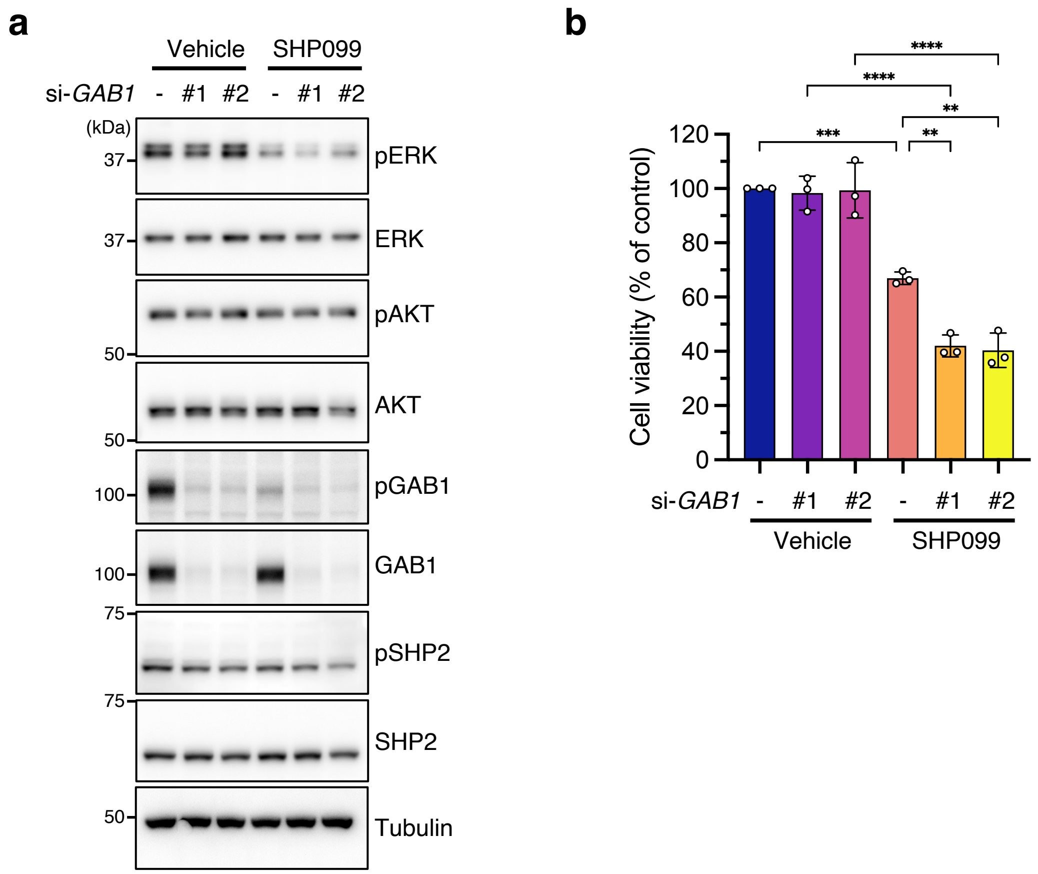

Fig. S6. Role of GAB1-SHP2 signaling in parental JMSU1 cells. a Western blot analysis of JMSU1 cells treated with *GAB1* siRNAs and SHP099. siRNA-transfected cells were treated with SHP099 (10 μM) or vehicle for 2 h before cell lysis. **b** Cell viability assay of J JMSU1 cells treated with *GAB1* siRNAs and SHP099. siRNA-transfected cells were treated with SHP099 (10 μM) or vehicle for 2 d, followed by a WST-8 assay (n = 3). Data are presented as mean ± SD; ***p*<0.01, ****p*<0.001, *****p*<0.0001; statistical significance determined by Tukey's test.

Supplementary Figure S7

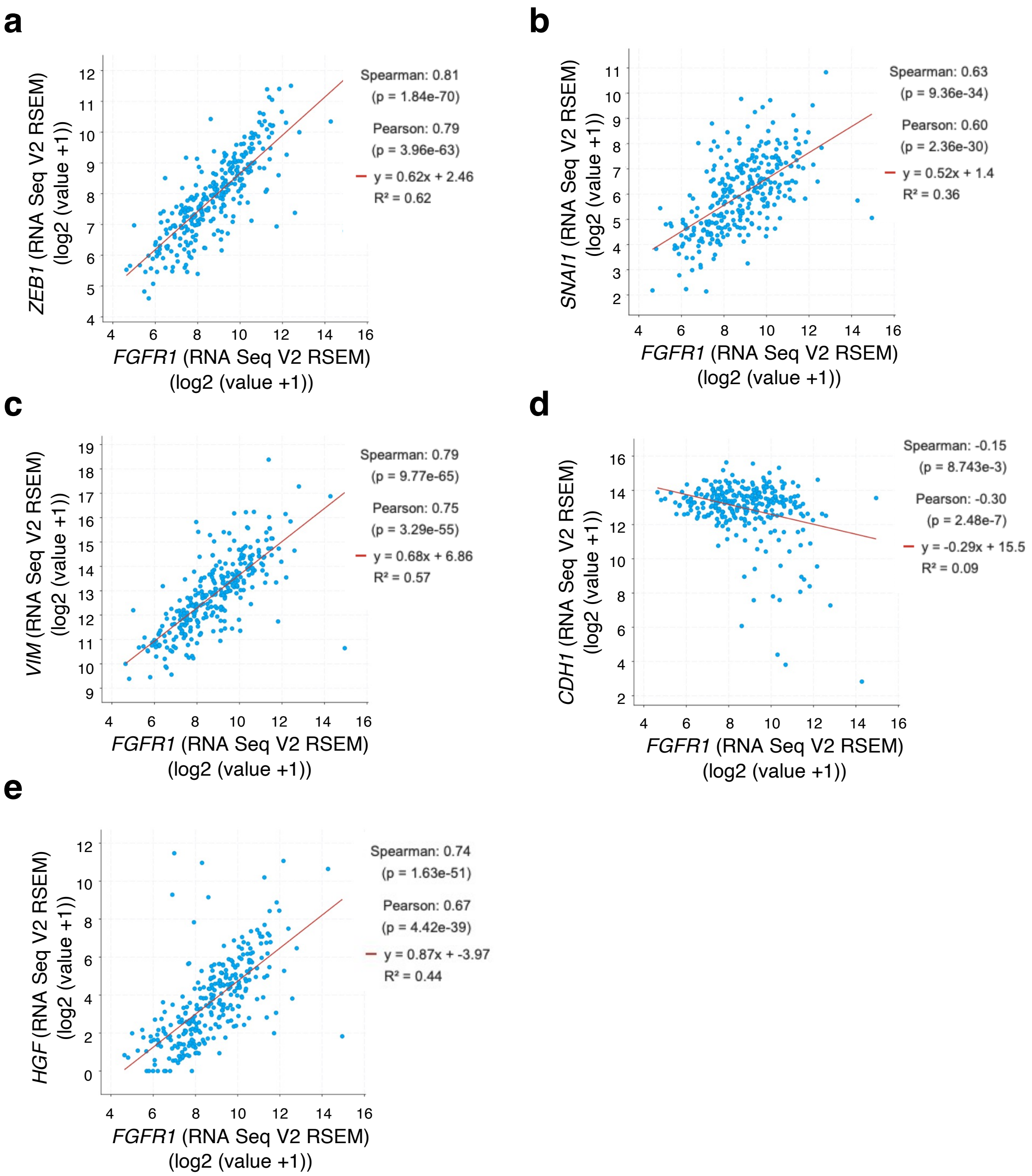

Fig. S7. TCGA data analysis of *FGFR1* mRNA expression correlation with EMT marker genes and *HGF* in MIBC. a-d Correlation between *FGFR1* mRNA expression and mesenchymal markers *ZEB1* (**a**), *SNAI1* (**b**), and *VIM* (**c**), as well as the epithelial marker *CDH1* (**d**). **e** Correlation between *FGFR1* and *HGF* mRNA expression.

Supplementary Figure S8

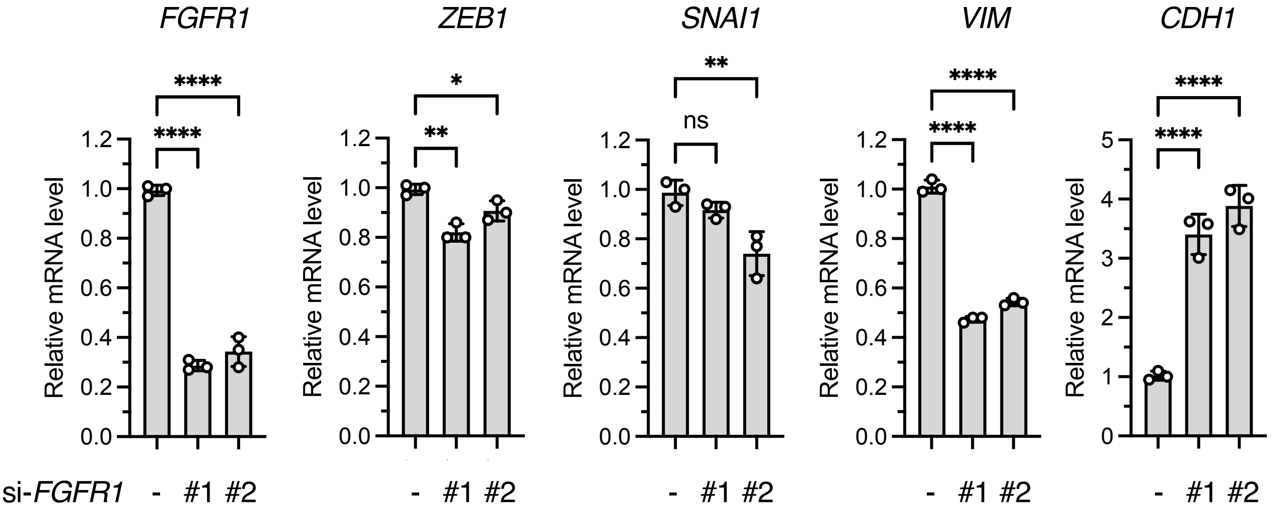

Fig. S8. Effect of *FGFR1* knockdown on EMT marker expression in JMSU1 cells. JMSU1 cells were treated with control (-) or *FGFR1* (#1, #2) siRNAs, and mRNA expression of the indicated genes was analyzed by quantitative RT-PCR (n = 3). Data are presented as mean ± SD; *p<0.05, **p<0.01, ****p<0.0001, ns, not significant; statistical significance determined by Dunnett's test.
