# Supplementary material for: MET signaling drives acquired resistance to erdafitinib in muscle-invasive bladder cancer cells
Source: Cell Death Dis. 2025 Nov 28;16(1):868. doi: 10.1038/s41419-025-08221-8 (PMC12663377; doi:10.1038/s41419-025-08221-8)
Supplement: Supplementary file 2 — Supplementary tables 1–3 [file 41419_2025_8221_MOESM2_ESM.docx]

**Supplementary Table 1. List of siRNAs**

| **Gene** | **Sequence (5′-3′) or siRNA ID** | **Source** |
| --- | --- | --- |
| *GAB1#1* | (sense) CCAAGAAGCCUAUUCGUAUTT  (antisense) AUACGAAUAGGCUUCUUGGTT | Sigma-Aldrich |
| *GAB1#2* | (sense) GCAGAUGAGAGAGUGGAUUTT  (antisense) AAUCCACUCUCUCAUCUGCTT | Sigma-Aldrich |
| *FGFR1#1* | (sense) CGGUCAUCGUCUACAAGAUTT  (antisense) AUCUUGUAGACGAUGACCGTT | Sigma-Aldrich |
| *FGFR1#2* | (sense) GAUGGUCCCUUGUAUGUCATT  (antisense) UGACAUACAAGGGACCAUCTT | Sigma-Aldrich |
| *MET* | SASI_Hs02_00329694 | Sigma-Aldrich |
| *-* | MISSION® siRNA Universal Negative Control #1(SIC001) | Sigma-Aldrich |

**Supplementary Table 2. List of antibodies for Western blotting**

| **Antigen** | **Source** | **Catalog no.** | **Dilution** |
| --- | --- | --- | --- |
| pAKT (Ser473) | Cell Signaling Technology | #4060 | 1/2000 |
| pERK1/2 (Thr202/Tyr204) | Cell Signaling Technology | #4370 | 1/2000 |
| pGAB1 (Tyr627) | Cell Signaling Technology | #3233 | 1/1000 |
| pMET (Tyr1234/1235) | Cell Signaling Technology | #3077 | 1/1000 |
| MET | Cell Signaling Technology | #3127 | 1/1000 |
| pSHP2 (Tyr542) | Cell Signaling Technology | #15543 | 1/1000 |
| STAT1 | Cell Signaling Technology | #14994 | 1/1000 |
| pSTAT3 (Tyr705) | Cell Signaling Technology | #9145 | 1/2000 |
| STAT3 | Cell Signaling Technology | #9139 | 1/1000 |
| AKT | Santa Cruz Biotechnology | sc-5298 | 1/1000 |
| ERK | Santa Cruz Biotechnology | sc-135900 | 1/1000 |
| FGFR1 | Santa Cruz Biotechnology | sc-57132 | 1/200 |
| GAB1 | Santa Cruz Biotechnology | sc-13391 | 1/200 |
| SHP2 | Santa Cruz Biotechnology | sc-7384 | 1/200 |
| pSTAT1 | Santa Cruz Biotechnology | sc-136229 | 1/200 |
| pEGFR (Tyr1092) | Proteintech | #30278-1-AP | 1/1000 |
| EGFR | MBL | MI-12-1 | 1/1000 |
| α-tubulin-HRP | MBL | PM054-7 | 1/10000 |
| **Secondary antibodies** | | | |
| Rabbit IgG-HRP | Bio-Rad | 1706515 | 1/10000 |
| Mouse IgG-HRP | Bio-Rad | 1706516 | 1/10000 |

**Supplementary Table 3. List of primers**

| **Gene** | **Forward primer (5′-3′)** | **Reverse primer (5′-3′)** |
| --- | --- | --- |
| *MET* | GCATTTTTACGGACCCAATC | TGTTCGATATTCATCACGGC |
| *HGF* | ACTGCAGACCAATGTGCTAATAGA | TGCTATTGAAGGGGAACCAG |
| *FGFR1* | GCTAAAGCACATCGAGGTGAATG | TCTCTTTGTCGGTATTAACTCC |
| *ZEB1* | GATGATGAATGCGAGTCAGATGC | ACAGCAGTGTCTTGTTGTTGT |
| *SNAI1* | GCCTTCAACTGCAAATACTGC | CTTCTTGACATCTGAGTGGGT |
| *VIM* | TGTCCAAATCGATGTGGATGTTTC | TTCTACCATTCTTCTGCCTCCTG |
| *CDH1* | CTGAGGATGGTGTAAGCGATG | GTCTGTCATGGAAGGTGCTC |
| *β-Actin* | CCCTGGAGAAGAGCTACGAG | TGAAGGTAGTTTCGTGGATGC |
