# Supplementary figures and images for: MET signaling drives acquired resistance to erdafitinib in muscle-invasive bladder cancer cells
Source: Cell Death Dis. 2025 Nov 28;16(1):868. doi: 10.1038/s41419-025-08221-8 (PMC12663377; doi:10.1038/s41419-025-08221-8)

Fig. 1h

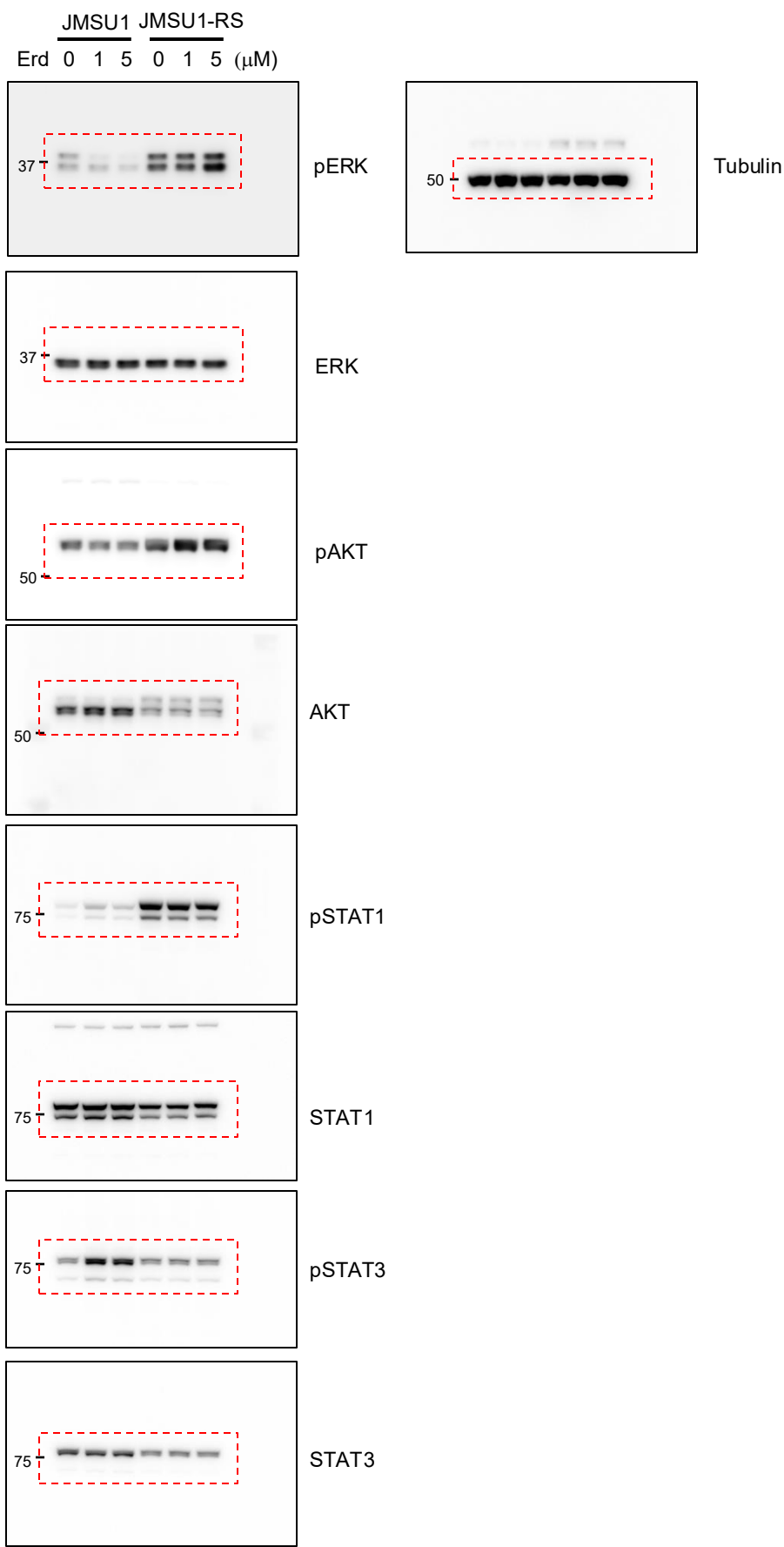

Fig. 2d

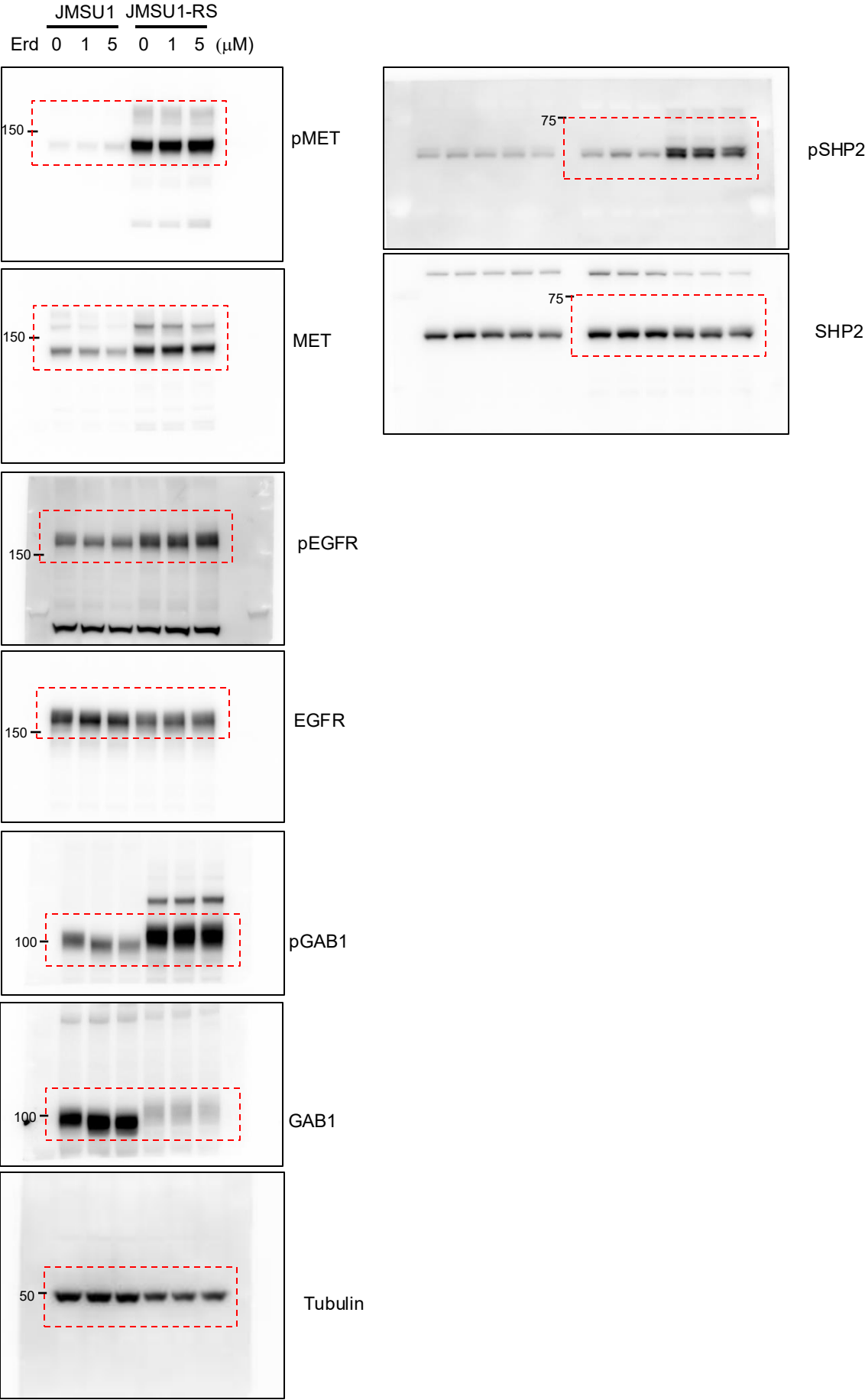

Fig. 3a

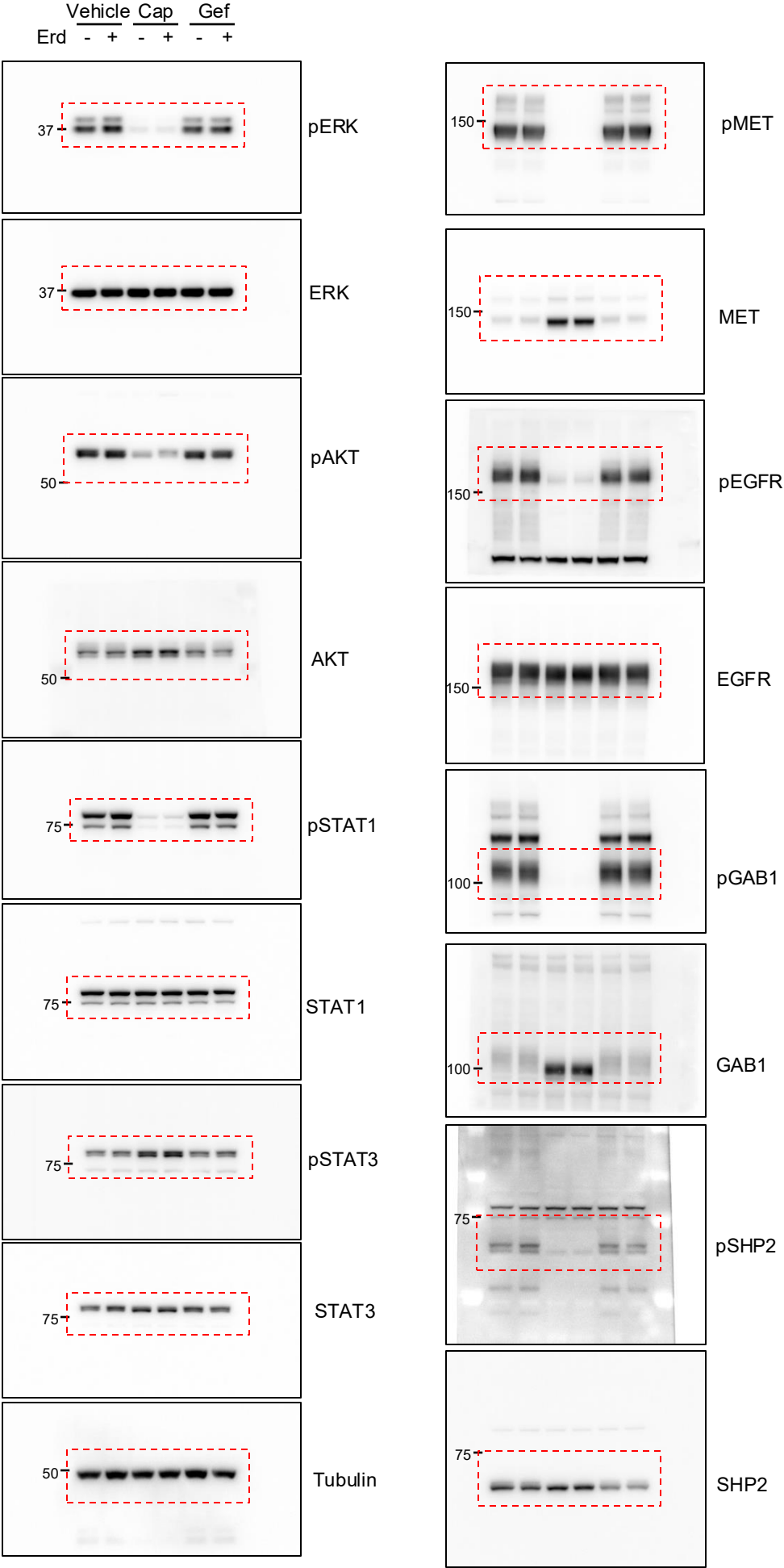

**Fig. 3b**

|          |   |   |   |   |
|----------|---|---|---|---|
| si-MET   | - | - | + | + |
| si-FGFR1 | - | + | - | + |

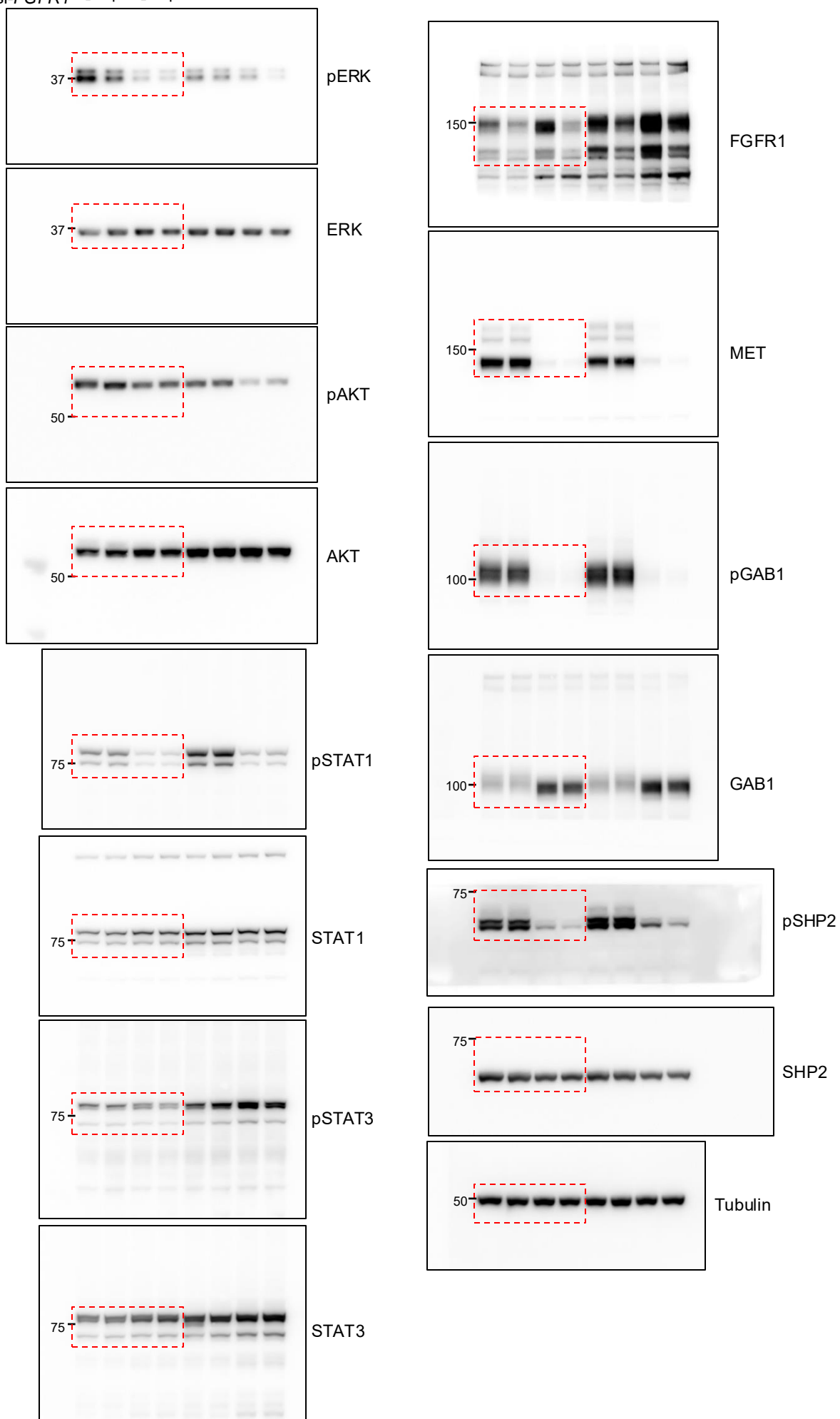

**Fig. 4c**

|     |   |   |   |   |        |
|-----|---|---|---|---|--------|
| Erd | 0 | 1 | 5 | 1 | 5 (μM) |
| HGF | - | - | - | + | +      |

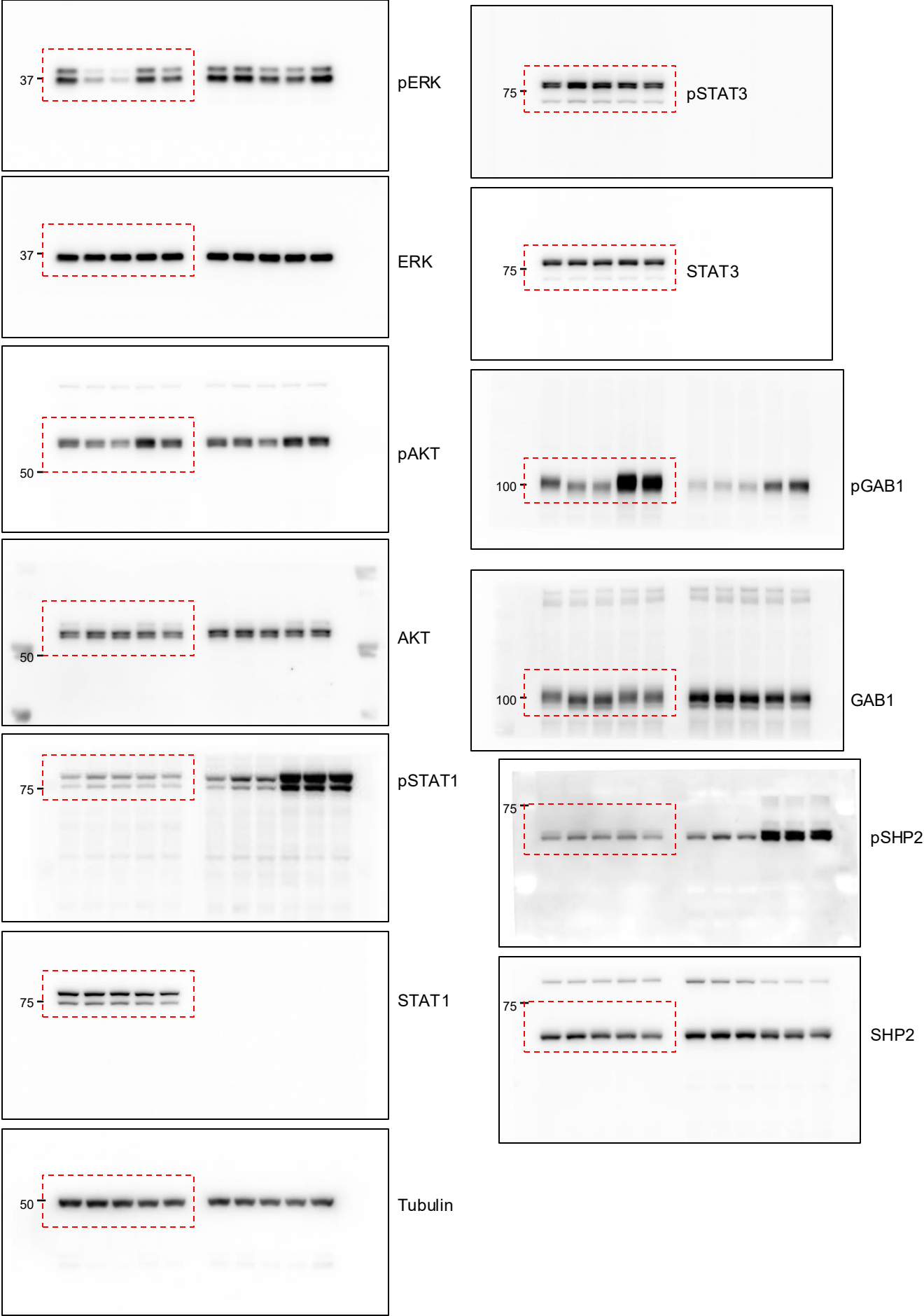

**Fig. 5c**

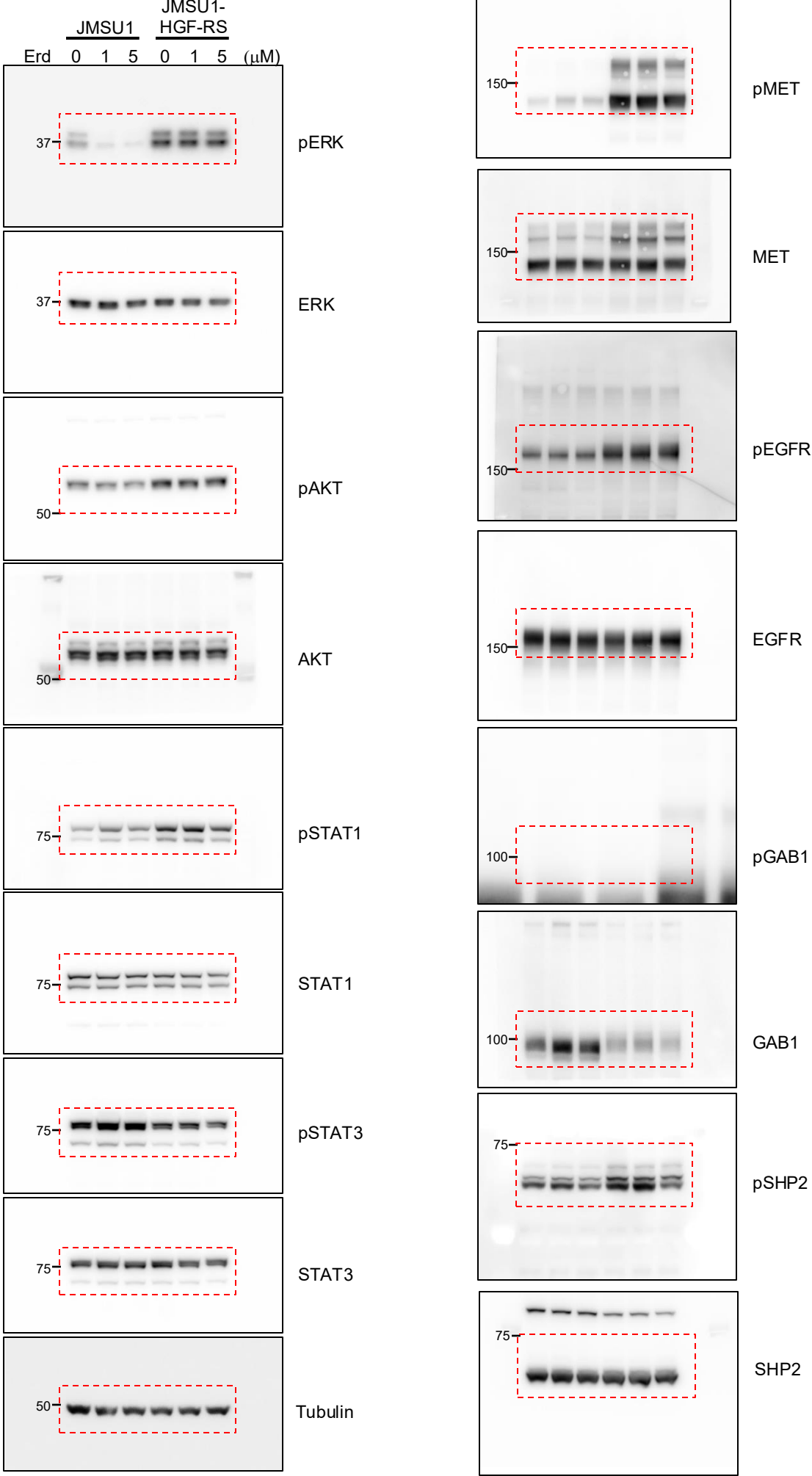

Fig. 6a

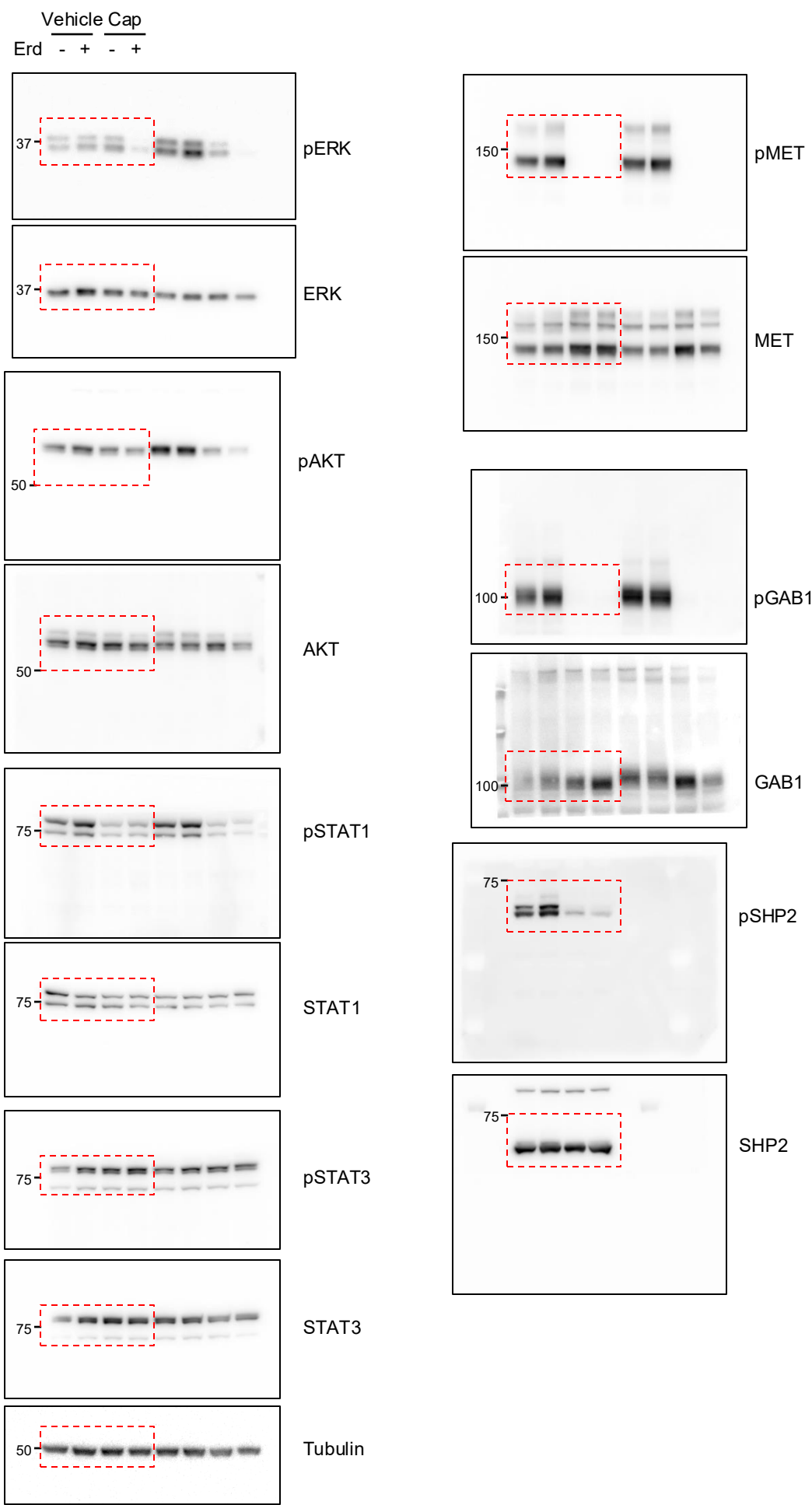

**Fig. 6b**

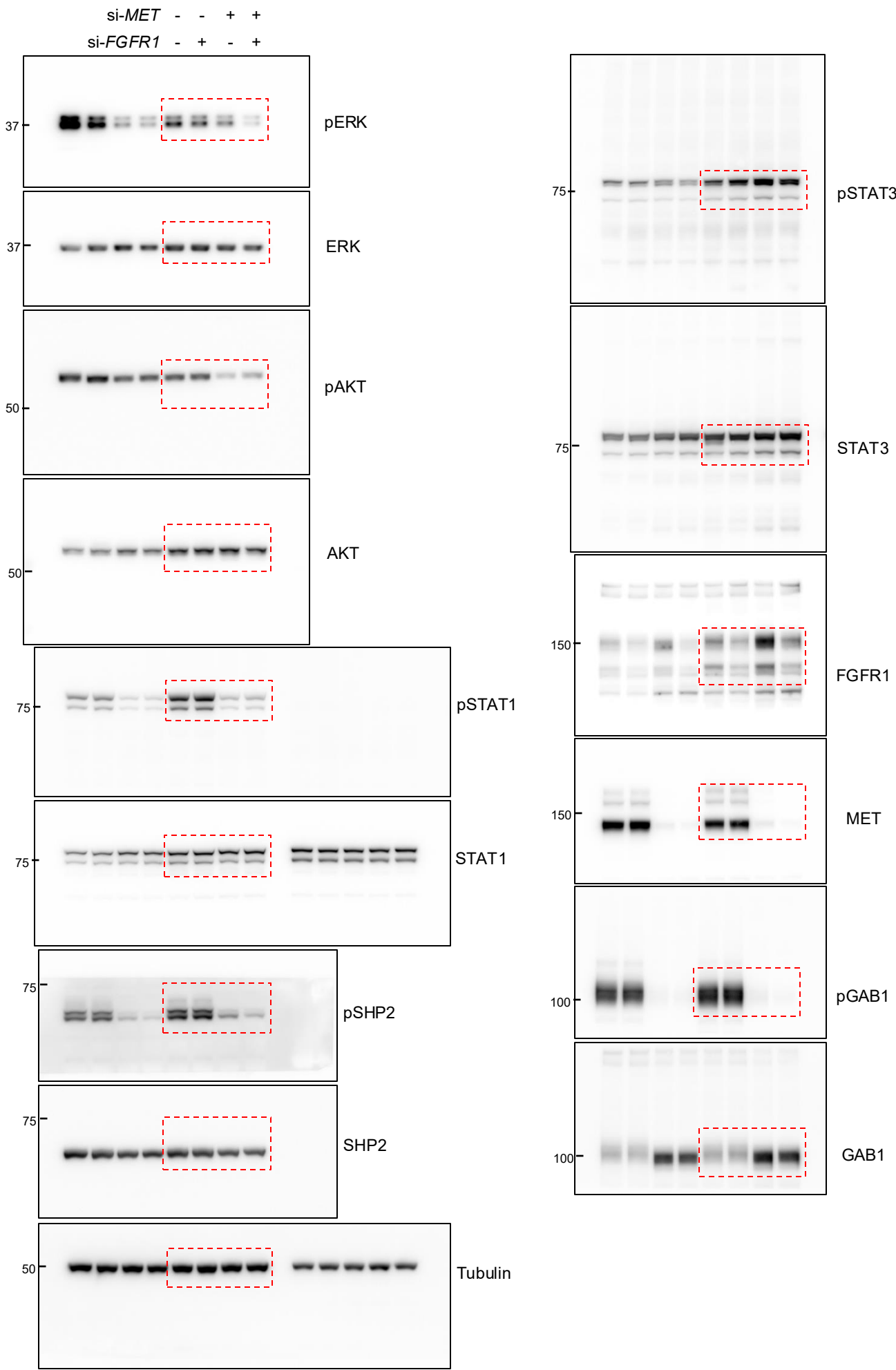

**Fig. 7a**

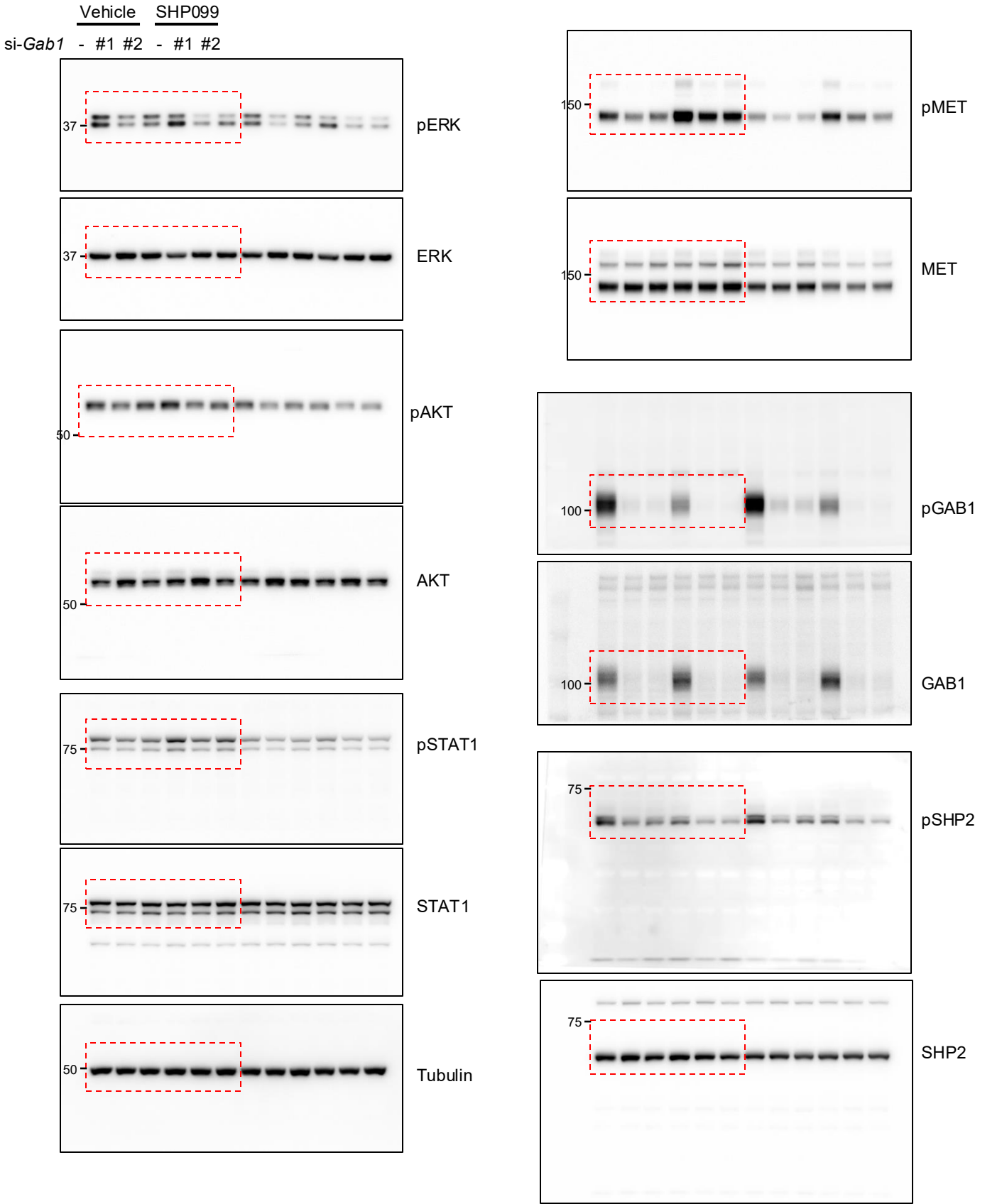

**Fig. 7b**

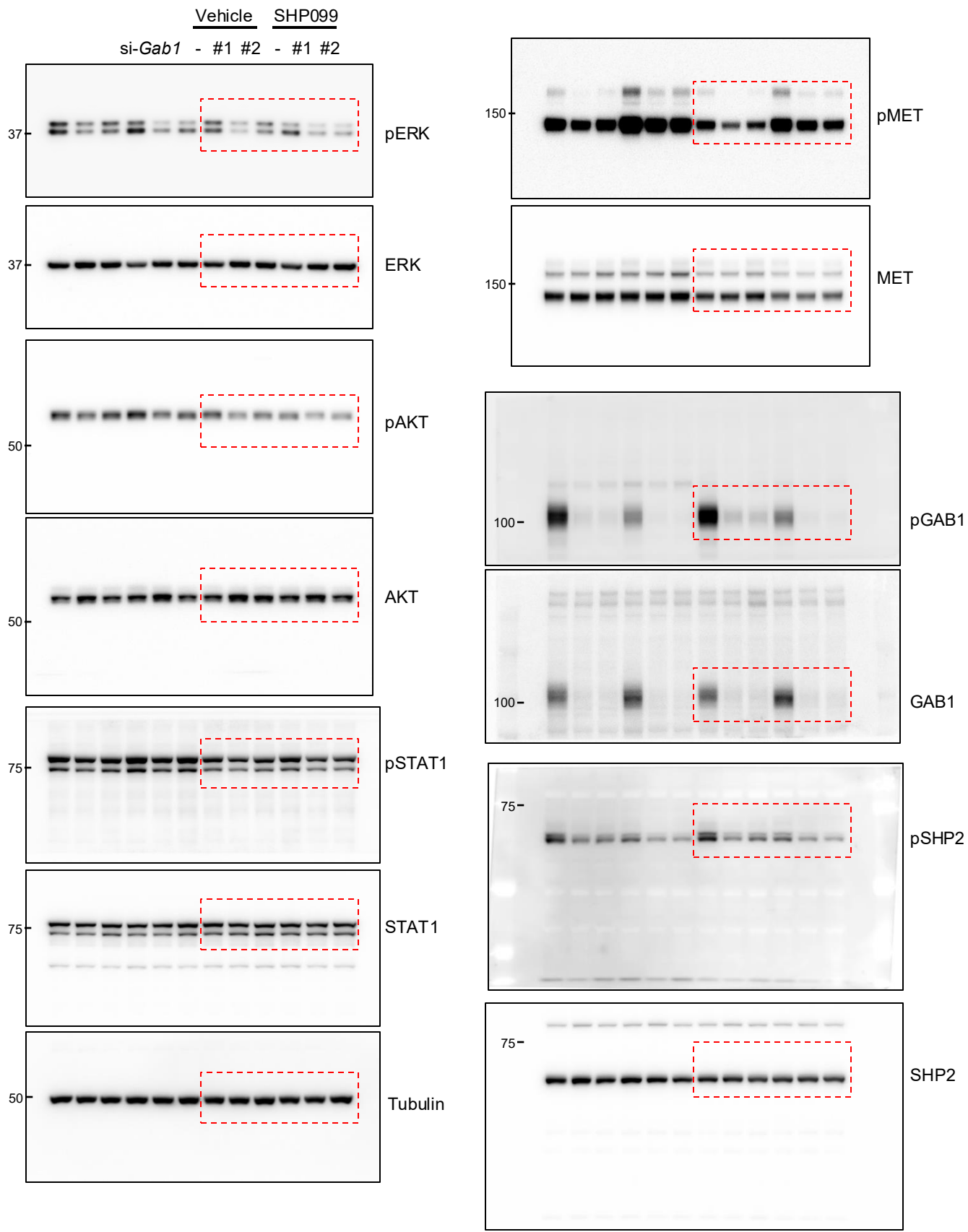

Fig. S2c

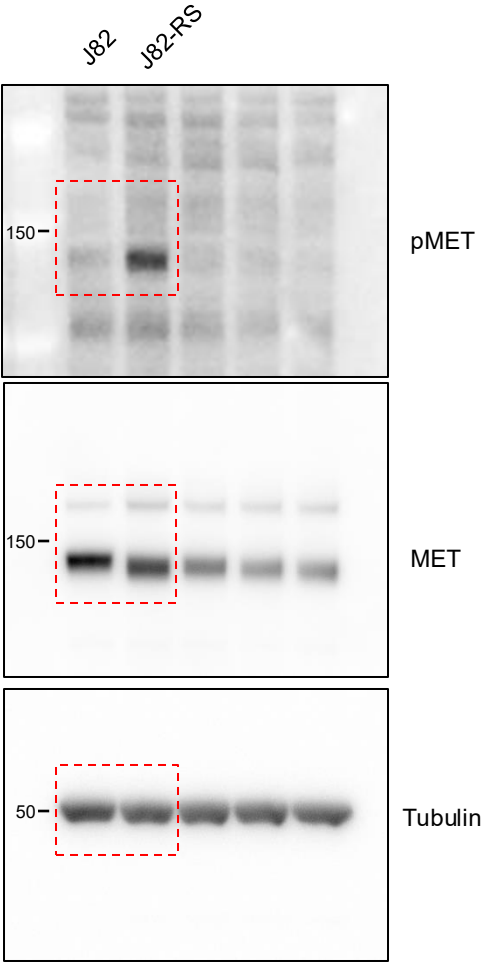

**Fig. S6a**

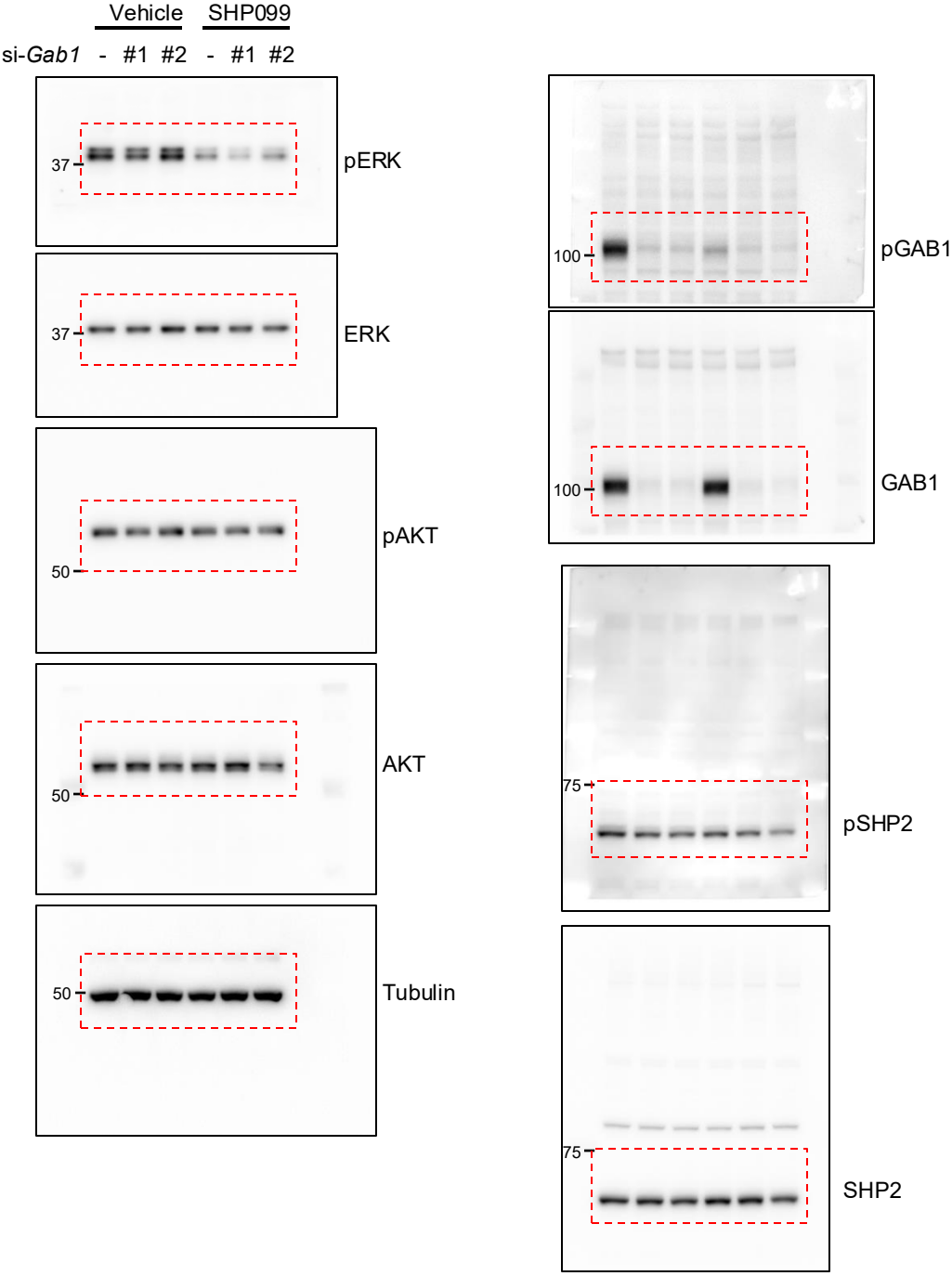

Supplement: Supplementary file 3 — Original Western blots [file 41419_2025_8221_MOESM3_ESM.pdf]
